# In silico assessment of biocompatibility and toxicity: molecular docking and dynamics simulation of PMMA-based dental materials for interim prosthetic restorations

**DOI:** 10.1007/s10856-024-06799-7

**Published:** 2024-06-04

**Authors:** Ravinder S. Saini, Rayan Ibrahim H. Binduhayyim, Vishwanath Gurumurthy, Abdulkhaliq Ali F. Alshadidi, Shashit Shetty Bavabeedu, Rajesh Vyas, Doni Dermawan, Punnoth Poonkuzhi Naseef, Seyed Ali Mosaddad, Artak Heboyan

**Affiliations:** 1 Department of Dental Technology, COAMS, King Khalid University, Abha, Saudi Arabia; 2 Department of Restorative Dentistry, College of Dentistry, King Khalid University, Abha, Saudi Arabia; 3 Department of Chemistry, Warsaw University of Technology, Warsaw, Poland; 4 Department of Pharmaceutics, Moulana College of Pharmacy, Kerala, India; 5 Department of Research Analytics, Saveetha Dental College and Hospitals, Saveetha Institute of Medical and Technical Sciences, Saveetha University, Chennai, India; 6 Student Research Committee, School of Dentistry, Shiraz University of Medical Sciences, Shiraz, Iran; 7 Department of Prosthodontics, Faculty of Stomatology, Yerevan State Medical University after Mkhitar Heratsi, Yerevan, Armenia; 8 Department of Prosthodontics, School of Dentistry, Tehran University of Medical Sciences, Tehran, Iran

## Abstract

Aim: This study aimed to comprehensively assess the biocompatibility and toxicity profiles of poly(methyl methacrylate) (PMMA) and its monomeric unit, methyl methacrylate (MMA), crucial components in dental materials for interim prosthetic restorations. Methodology: Molecular docking was employed to predict the binding affinities, energetics, and steric features of MMA and PMMA with selected receptors involved in bone metabolism and tissue development, including RANKL, Fibronectin, BMP9, NOTCH2, and other related receptors. The HADDOCK standalone version was utilized for docking calculations, employing a Lamarckian genetic algorithm to explore the conformational space of ligand-receptor interactions. Furthermore, molecular dynamics (MD) simulations over 100 nanoseconds were conducted using the GROMACS package to evaluate dynamic actions and structural stability. The LigandScout was utilized for pharmacophore modeling, which employs a shape-based screening approach to identify potential ligand binding sites on protein targets. Results: The molecular docking studies elucidated promising interactions between PMMA and MMA with key biomolecular targets relevant to dental applications. MD simulation results provided strong evidence supporting the structural stability of PMMA complexes over time. Pharmacophore modeling highlighted the significance of carbonyl and hydroxyl groups as pharmacophoric features, indicating compounds with favorable biocompatibility profiles. Conclusion: This study underscores the potential of PMMA in dental applications, emphasizing its structural stability, molecular interactions, and safety considerations. These findings lay a foundation for future advancements in dental biomaterials, guiding the design and optimization of materials for enhanced biocompatibility. Future directions include experimental validation of computational findings and the development of PMMA-based dental materials with improved biocompatibility and clinical performance.

**Graphical Abstract:**

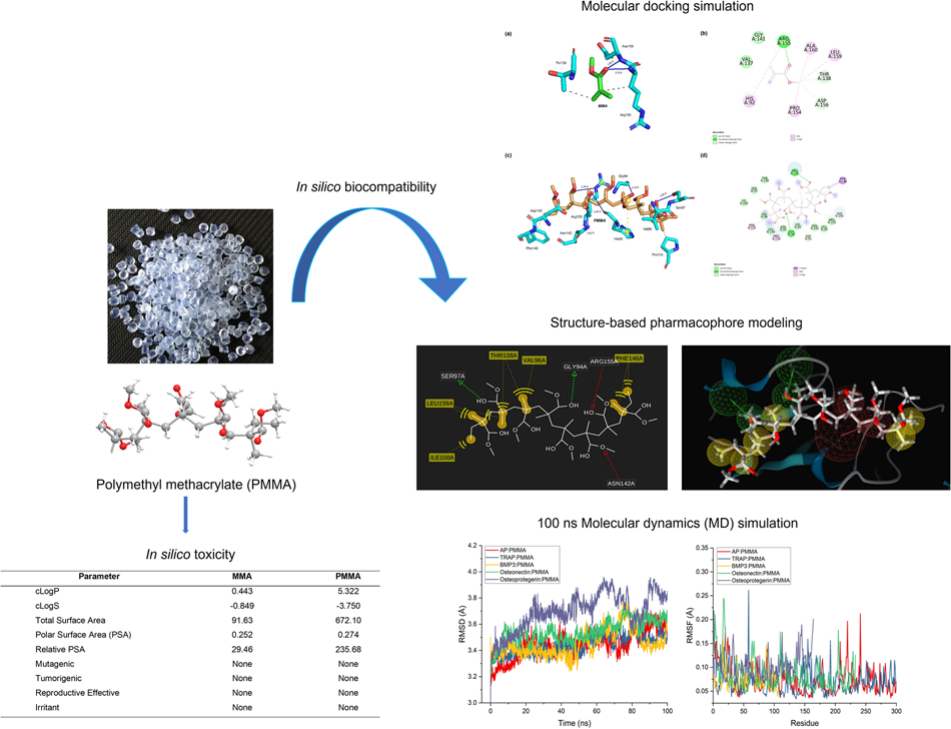

## Introduction

Interim prosthetic restorations play a vital role in dental care by providing temporary solutions for missing or damaged teeth until permanent prostheses can be fabricated. These interim restorations serve as a crucial intermediate step in dental treatment, offering patients both esthetic and functional benefits [1, 2]. They are particularly important in various clinical scenarios, such as the period between final impressions and the delivery of permanent prostheses, following dental implant surgery, or during the phase preceding the fitting of final abutments. Additionally, interim restorations are utilized following extended restorative treatments when a patient’s oral function and esthetics may be compromised. These restorations not only provide essential oral support but also contribute to maintaining esthetics, which can significantly impact a patient’s psychosocial well-being. Acting as a temporary solution for a limited duration, interim restorations are eventually replaced by permanent prostheses at a later stage. This seamless transition enables the continued functionality of the oral cavity while ensuring optimal esthetics and patient satisfaction [3–6].

Polymethyl methacrylate (PMMA), a synthetic polymer based on the monomer methyl methacrylate (MMA), is one of the main materials used to make provisional restorations and is part of a typical workflow in prosthodontics. PMMA is popular in the dental field because it is suitable for short-term interim restorations in the oral cavity. These properties include excellent biocompatibility, low cytotoxicity, and ease of handling [7–9]. It is used as a provisional biomaterial owing to exceptional compatibility with biological systems. PMMA causes little or no irritation or inflammation in oral tissues; thus, patients can be treated during the intermediate treatment phase with PMMA-based materials without being subjected to undue tissue discomfort and disruption [10, 11]. Furthermore, the ease with which PMMA can be handled allows for quick chairside clinical manipulation, which benefits clinicians and patients [8, 12]. Despite the tremendous versatility and excellent inertness of PMMA, concerns regarding its biocompatibility and toxicity in dental applications have been raised. Biocompatibility is a crucial concern for any dental material because it ensures that it does not generate an external or local response to a physiological system, including the local tissues (gingiva, bone, and periodontium) around the restoration and adjacent teeth. This is a significant aspect that must be considered. Biocompatibility depends on the release of ions from the material, its surface characteristics, and its interactions with living tissues [13, 14]. As it is in the oral environment, it is essential to assess its safety profile. This assessment is important to determine the risks of using PMMA-based dental materials on patients. Therefore, clinicians might appreciate a detailed account of the biocompatibility [15] and toxicity of this type of material in dental clinics to navigate patients’ concerns and decisions better [16]. Furthermore, toxicity assessments are essential for evaluating dental materials [17]. Although the toxicity of PMMA is low, some of its constituents or degradation products may pose a risk to oral tissues. Several studies have highlighted toxicity risks associated with constituents and degradation products of PMMA. Additives such as Bisphenol-A (BPA) and its derivatives, used in PMMA production, pose concerns due to their endocrine-disrupting properties [18, 19].

To address these concerns, this study utilized modern in silico analytical approaches, including molecular docking and molecular dynamics simulations, to evaluate the biocompatibility and toxicity of dental materials based on PMMA. Traditional evaluation methods for the biological or biomedical compatibility and toxicity of dental materials have limitations in providing molecular-level details of the interactions between the tested materials and biological systems [20–22]. With the development of various in silico techniques, such as molecular docking and molecular dynamics, it is possible to gain a more detailed understanding of molecular interactions between dental materials and biological tissues [23, 24]. Furthermore, this in silico approach enabled researchers to systematically address previously unanswered questions regarding the molecular interactions between PMMA-based dental materials and biological targets, including the likely binding sites, affinities, and structural variations over time. Furthermore, rapid in silico screening could help recognize potential hazards in dental materials, and this could promote better and more informed decisions regarding material selection for temporarily substituting lost natural teeth for interim prosthetic restorations. Using in silico approaches, we aimed to provide an understanding of the behavior of a material at the level of macromolecular interactions in its environment, particularly with biological entities [25, 26]. A more predictive understanding of the in vivo behavior of PMMA-containing dental materials, potentially leading to biocompatibility and toxicity assessments, can be obtained more systematically and cost-effectively. Previous studies have investigated the biocompatibility and toxicity of dental materials [17, 27, 28]. However, these studies often had limitations, such as a lack of molecular-level understanding of interactions between the materials and biological systems. Additionally, few studies have comprehensively evaluated the safety profile of PMMA-based materials using in silico approaches [29, 30]. Therefore, there was a gap in the literature regarding the detailed molecular analysis of PMMA biocompatibility and toxicity, which the present study aimed to address. By employing modern in silico analytical techniques, this study aimed to investigate the binding affinities and potential toxicity risks associated with MMA and PMMA in interactions with receptors involved in bone metabolism and tissue development. By employing molecular docking, molecular dynamics simulations, and pharmacophore modeling, we aimed to elucidate the molecular mechanisms underlying the interactions between these materials and selected receptors. Furthermore, we sought to identify active functional groups contributing to biocompatibility and assess the potential toxicity of PMMA.

## Methodology

This study aimed to assess the biocompatibility and toxicity profile of PMMA, a dental resin, and its precursor monomer MMA. To achieve this goal, we employed a systematic approach. First, we selected a specific set of receptors known to be relevant to dental biocompatibility as molecular docking targets. These receptors were constructed based on their experimental 3D structures, which were then energy-minimized to obtain their final conformations. Subsequently, we conducted molecular docking studies using MMA and PMMA to elucidate the molecular interactions and dynamic behaviors resulting from the interfacial interactions. Additionally, we employed various in silico tools for toxicity evaluations to predict potential toxic elements or compounds.

### Selection of receptors (proteins) related to MMA and PMMA biocompatibility

In this section, we discuss the methodology for selecting receptors or proteins relevant to the biocompatibility of MMA and PMMA in the oral environment. The rationale behind this selection process is outlined, emphasizing the importance of considering various factors such as bone metabolism, tissue interaction, and inflammatory responses. The selection of receptors involved a comprehensive assessment of their roles in the oral microenvironment, with a focus on bone metabolism and tissue interaction due to the common use of PMMA-based materials in dental applications. Attention was also given to receptors implicated in inflammatory responses, considering the potential immune reactions triggered by dental materials. Receptors that had been previously studied or published in the context of dental materials were prioritized for comparative analysis. The 3D structures of selected receptors were obtained from reputable databases such as the Protein Data Bank (PDB) [31] and AlphaFold [32]. Computational analyses were then conducted to identify the active sites of these receptors with maximum precision. CASTp 3.0 [33] was utilized for this purpose, allowing for the accurate identification of active sites crucial for ligand binding. The careful curation of receptors based on their roles in bone metabolism, tissue interaction, and inflammatory processes provided a solid foundation for investigating the biological activity of PMMA within the oral microenvironment. These selected receptors served as targets for molecular docking simulations, enabling the exploration of interactions between MMA/PMMA and specific biological targets relevant to dental biocompatibility.

### 3D Structure modeling of MMA and PMMA with MM2 energy minimization

In this part, we describe the methodology used to construct three-dimensional (3D) molecular structures of MMA and PMMA, followed by energy minimization to optimize their geometries. The rationale for employing these techniques is elucidated, emphasizing the importance of accurately representing ligand structures for subsequent molecular docking studies. The 3D molecular structures of MMA and PMMA were initially constructed to mimic the spatial arrangement of atoms, ensuring a realistic depiction of ligand-receptor interactions. To enhance accuracy, these structures underwent re-optimization using Chem3D molecular modeling software [34]. Additionally, an energy minimization step was performed using the MM2 force field [35, 36] in ChemDraw Professional 20.1.1 (PerkinElmer Inc.). This dual approach aimed to stabilize the ligand structures and eliminate any unfavorable conformations, thereby facilitating robust and reliable molecular docking simulations. Chem3D molecular modeling software, provided by PerkinElmer Inc., was utilized for constructing and optimizing the 3D structures of MMA and PMMA. This software offers advanced capabilities for accurately representing molecular geometries and performing energy minimization. The MM2 force field, integrated into ChemDraw Professional, was employed for energy minimization, leveraging its ability to calculate molecular energies and optimize molecular conformations. The 3D structures of MMA and PMMA, obtained through meticulous modeling and energy minimization, served as starting points for molecular docking simulations. By ensuring the stability and accuracy of these ligand structures, we aimed to obtain reliable insights into their interactions with target receptors related to dental biocompatibility. This rigorous ligand preparation process was crucial for conducting subsequent analyses of ligand-receptor binding affinities and interaction patterns.

### Molecular docking simulation

This section outlines the methodology employed for molecular docking simulations, highlighting the tools utilized and their significance in elucidating the interactions between MMA and PMMA with selected target receptors. Molecular docking serves as a pivotal technique for investigating the binding affinities and interaction patterns of ligands with receptor proteins. Molecular docking simulations were conducted using the HADDOCK standalone version [37], chosen for its advanced interface and capability to perform protein-ligand docking simulations with high precision. This tool facilitates the exploration of complex interactions between MMA and PMMA and their respective target receptors. The simulations were guided by specific criteria, including the identification of significant clusters or populations and the determination of docking scores, which serve as indicators of binding affinity. Additionally, binding affinities were predicted using PRODIGY [38] to further enrich the theoretical results and provide insights into the thermodynamic features of protein-ligand interactions. The HADDOCK standalone version is a widely used software tool for performing protein-ligand docking simulations, renowned for its accuracy and versatility in capturing intricate molecular interactions. PRODIGY, on the other hand, is employed to predict binding affinities and analyze the thermodynamic properties of protein-ligand complexes, thereby complementing the insights obtained from molecular docking simulations. In this study, molecular docking simulations were instrumental in elucidating the interactions between MMA and PMMA with target receptors related to dental biocompatibility. By incorporating the intrinsic flexibility of both ligands and receptors, these simulations provided detailed insights into binding affinity, potential binding sites, and atomic-level interactions [39, 40]. The results of these simulations laid the groundwork for understanding the molecular dynamics and biocompatibility implications of MMA and PMMA, thus contributing to a comprehensive assessment of their suitability for dental applications.

### Pharmacophore modeling to assess active functional groups of MMA and PMMA

This section outlines the utilization of pharmacophore modeling to identify critical functional groups of MMA and PMMA at the molecular level. Pharmacophore modeling serves as a crucial technique for elucidating the key molecular elements involved in ligand-receptor interactions, thereby shedding light on the molecular determinants of biocompatibility. Pharmacophore modeling was performed using LigandScout 4.5 [41], a sophisticated software tool designed for the identification and characterization of key pharmacophoric elements in ligands. This technique enables the systematic exploration of molecular interactions and the identification of active functional groups crucial for ligand-receptor interactions. LigandScout 4.5 is a widely used software platform known for its advanced capabilities in pharmacophore modeling and ligand-based virtual screening. It allows for the efficient identification and characterization of pharmacophoric features essential for molecular recognition, making it an ideal tool for assessing the active functional groups of MMA and PMMA. In this study, pharmacophore modeling was employed to delineate the key pharmacophoric elements of MMA and PMMA, providing insights into their interactions with biological receptors. By identifying the active functional groups that contribute to biocompatibility, pharmacophore modeling facilitated a nuanced understanding of the molecular mechanisms underlying the interactions between MMA, PMMA, and biological receptors. This information was crucial for assessing the biocompatibility of MMA and PMMA-based dental materials and elucidating their suitability for clinical applications.

### Molecular dynamics (MD) simulation

This section describes the implementation of MD simulation as a computational technique to investigate the dynamic behavior of ligand-receptor complexes, specifically focusing on MMA and PMMA molecules within the oral environment. MD simulation enables the exploration of structural stability, flexibility, and conformational changes over extended timescales, providing valuable insights into the behavior of these molecules. MD simulations were conducted using GROMACS 2023.3 [42], a widely used software package for molecular dynamics simulations. The choice of GROMACS was based on its robustness, efficiency, and extensive features tailored for simulating complex biomolecular systems. The General Amber Force Field (GAFF2) [43] was selected to parameterize the ligands (MMA and PMMA), ensuring an accurate representation of their interactions within the simulation environment. Partial charges for the ligands were obtained using the Austin Model 1 semi-empirical molecular orbital technique combined with Bond Charge Correlations (AM1-BCC) [44], which enhances the accuracy of electrostatic interactions. GROMACS 2023.3 is equipped with a suite of force fields, integration algorithms, and analysis tools optimized for MD simulations of biomolecular systems. Its versatility and performance make it an ideal choice for studying the dynamic behavior of ligand-receptor complexes. The GAFF2 force field provides parameters for a wide range of organic molecules, ensuring a reliable representation of non-covalent interactions in the simulation. The protein simulations utilized the AMBER99sb force field [45], and the simulation box included SPC water and neutralizing counterions. Short-range non-bonded interactions were truncated at 2.0 nm, and long-range electrostatics were computed using the Particle Mesh Ewald (PME) method [46]. The simulation protocol included first minimizing with the steepest descent procedure to a point where the maximum force of the system was under 1000 kJ/mol/nm, then a 1000 ps restrained NVT simulation, and a 1000 ps restrained NPT simulation. The starting stage included a Berendsen thermostat at 310 K and a Berendsen barostat at 1 bar. The final phase included an unconstrained 100 ns simulation, including the Berendsen thermostat and Parrinello-Rahman barostat. This long simulation provided a detailed interaction between the ligand and receptor in a dynamic view of MMA and PMMA behavior in a simulated oral environment for more extended periods. The different environmental conditions and simulation steps provided robust and detailed information about the behavior of the polymers in their oral environment over a longer time frame. By subjecting the ligand-protein complexes to MD simulations, we gained detailed insights into their interactions, structural stability, and conformational changes over time. This information provided a dynamic view of MMA and PMMA behavior in the simulated oral environment, offering valuable insights for assessing their biocompatibility and toxicity over extended periods.

### In silico toxicity assessment of MMA and PMMA

This section outlines the crucial phase of the methodology focused on assessing the safety profile of MMA and PMMA using state-of-the-art in silico tools. The rationale behind employing these tools lies in their ability to develop mathematical models based on molecular descriptors of the compounds, enabling toxicity assessments. By leveraging complex algorithms, these tools predict the presence of potentially toxic elements or compounds in the molecular structures of MMA and PMMA, thereby contributing to a comprehensive understanding of their toxicological potential. Two main in silico tools were utilized for toxicity assessment: OSIRIS DataWarrior V5.5.0 [47] and the QikProp [48] module of the Schrödinger Software Suites. OSIRIS DataWarrior V5.5.0 offers a comprehensive database and prediction tools that facilitate the assessment of MMA and PMMA’s toxicological potential. It evaluates various molecular characteristics to identify indicators of toxicity risk, thereby providing valuable insights into potential toxicological implications. Additionally, the QikProp module of the Schrödinger Software Suites employs advanced algorithms to analyze molecular properties and predict potentially toxic elements or compounds. By integrating these tools into the toxicity assessment, a robust evaluation of the safety profiles of MMA and PMMA in dentistry is achieved. In this study, the toxicity assessment phase plays a crucial role in comprehensively evaluating the safety profiles of MMA and PMMA. By developing mathematical models based on molecular descriptors and leveraging cutting-edge algorithms, OSIRIS DataWarrior V5.5.0 and the QikProp module predict potential toxicological risks associated with the compounds. These predictions enhance our understanding of the safety implications of using MMA and PMMA in dental applications. By considering toxicity assessment as an integral part of the study, a holistic perspective on the potential risks associated with MMA and PMMA usage in dentistry is achieved, thereby informing decision-making processes and ensuring patient safety.

## Results and discussion

### Selection of receptors (proteins) related to MMA and PMMA biocompatibility

Table 1 reports the biocompatibility receptors (proteins) we selected in connection with MMA and PMMA and the findings. These receptors are involved in crucial bone metabolism, tissue development, and cellular processes related to dental and bone homeostasis. These were selected because they are possible targets of Bone morphogenetic proteins (BMPs) BMP2, BMP3, BMP7, and BMP9 were chosen as targets because these growth factors act as crucibles for bone formation and regeneration [49, 50]. In particular, BMP2 and BMP7 play crucial roles in osteogenesis, making them two of the most important targets for the evaluation of PMMA-based precursors for biocompatibility [51, 52]. These BMPs have active sites, as described in the literature, such as residues 293–336 in BMP2 [53]. The list of residues in the active sites could help us understand the PMMA-binding regions essential for interaction with the dental material. Because BMPs play an important role in osteogenicity, the interaction of PMMA with BMPs shed light on their interference in osteogenic reactions and the osseointegration process. COL1A1, a vital component of bone extracellular matrix [54], was chosen to explore the interaction between PMMA-based materials and the structural framework of the bone. The selected active sites, including residues 50–71, signify the regions crucial for maintaining the integrity and stability of the bone matrix [54]. Investigating how PMMA interacts with COL1A1 offers valuable insights into its potential effects on bone structure and strength. Dentin matrix acidic phosphoprotein 1 (DMP-1), associated with dentin formation and mineralization [55], was used to investigate the interaction of PMMA-based materials with proteins involved in dentinogenesis. How PMMA influences DMP-1 sheds light on its impact on dental tissues, mineralized structures, and overall oral health.

**Table 1:** The results related to the preparation of target proteins were retrieved from the RCSB PDB, and the active sites were determined using CASTp 3.0

Name	PDB / UniProt ID	Resolution (Å)	Chain	Weight (kDa)	Sequence Length	Active Site (Residue number)
AP	2GLQ [64]	1.60	A	53.57	484	15, 18, 19, 22, 68, 72, 73
BMP2	4UI1 [53]	2.35	A	51.34	114	293, 295, 296, 328, 329, 330, 331, 332, 333, 334, 335, 336, 338, 339, 344, 347, 348, 350, 351, 358, 393, 394, 395
BMP3	2QCQ [65]	2.21	A	24.84	110	4, 37, 38, 39, 40, 41, 58, 62, 69, 71, 72, 73, 74, 75, 76, 77, 110
BMP7	1LX5 [66]	3.30	A	29.23	139	47, 48, 53, 54, 55, 56, 57, 58, 59, 60, 117
BMP9	1ZKZ [67]	2.33	A	12.1	110	40, 41, 42, 43, 59, 62, 63, 71
COL1A1	5CTD [68]	1.60	C	21.06	72	50, 53, 54, 57, 70, 71
DMP-1	A0A804HJB8 [69]	N/A	N/A	7.14	65	18, 19, 20, 21, 22, 23, 28, 30, 31, 34, 39, 40, 41, 42, 43, 44, 45, 46, 47
Fibronectin	1FNH [70]	2.80	A	29.63	271	101, 102, 131, 156, 158, 159, 160, 177, 178, 179, 180, 257, 259, 260
IGF-1	2DSP [71]	2.50	B	17.49	70	12, 15, 16, 17, 18, 19, 20, 21, 22, 23, 54, 64
NOTCH2	2OO4 [72]	2.00	A	52.97	234	1462, 1478, 1485, 1490, 1495, 1500, 1525, 1532, 1541, 1574, 1630, 1645, 1658
Osteocalcin	1Q8H [73]	2.00	A	5.85	49	16, 19, 38, 39, 42, 43
Osteonectin	1BMO [74]	3.10	A	55.23	233	65, 67, 77, 78, 79, 80, 81, 82, 83, 103, 107, 110, 111, 114, 115, 120
Osteopontin	1MOY [75]	1.55	A	13.80	130	64, 65, 67, 68, 69
Osteoprotegerin	3URF [76]	2.70	B	38.38	171	9, 10, 22, 29, 32, 48, 49, 50, 51, 52, 53, 54, 57, 58, 60, 64, 67, 70, 81, 82, 83, 85, 92, 116
Osterix	6×46 [77]	N/A	A	14.35	121	17, 23, 33, 37, 51, 57, 67, 71, 76
RANKL	3URF [76]	2.70	A	35.48	162	180, 181, 241, 245, 252, 257, 284, 287, 292
RUNX2	6VGD [78]	4.20	D	59.70	177	117, 164, 165, 200, 210, 212
TGF-β1	5VQP [79]	2.90	A	42.08	363	26, 29, 58, 72, 73, 74, 75, 77, 119, 120, 121, 122, 123, 170, 172, 176, 234, 239, 242, 254, 261, 298, 313, 321, 357, 361
TRAP	1WAR [80]	2.22	A	35.48	310	92, 93, 94, 95, 96, 97, 100, 114, 115, 137, 138, 141, 142, 145, 146, 153, 154, 155, 156, 159, 163
Wnt3	6AHY [81]	2.80	B	39.65	319	75, 78, 81, 85, 92, 228, 298, 299, 322, 349, 351, 353

IGF-1, responsible for generating a range of biochemical signals that enhance adult bone growth and regeneration [56], was selected as a marker of growth-associated pathways. The mechanisms by which PMMA interacts with IGF-1 provide insights into the possible effects of these materials on bone development, regeneration, and dental health. NOTCH2, a receptor involved in lineage specification and tissue morphogenesis [57], was used to investigate the role of PMMA-based materials in cell differentiation processes. Examining the interactions with NOTCH2 provides insights into its potential effects on tissue development, homeostasis, and oral health. Receptor Activator of Nuclear Factor Kappa-B Ligand (RANKL), a key regulator of osteoclast differentiation and bone remodeling [58], was chosen to investigate the influence of PMMA-based materials on bone resorption processes. Understanding how it interacts with RANKL can provide key indications of its possible effects on bone remodeling and bone health. Transforming Growth Factor Beta 1 (TGF-β1), a growth factor with multifaceted roles in tissue development and repair [59], was chosen to study the potential of PMMA-based biomaterials to alter TGF-β1-mediated cellular responses. Studying these interactions may provide insights into their potential effects on tissue development, repair, and oral health. The Wnt3 wingless-type MMTV integration site family signal was also selected because it is essential for the elaboration of an organism’s tissues, especially for bone formation [60]. The active site (residues 75–353) was chosen because it represents regions shown to be important in Wnt3-mediated events. This choice of the active site provides insights into the potential effects on tissue development, homeostasis, and overall oral health.

The resolution, chain information, weight, sequence length, and active site information for each selected receptor are listed in detail. These receptors constitute a diversity representing different aspects of bone and tissue biology. A comprehensive data-based catalog of the biocompatibility evaluation of PMMA-based dental materials was covered. In the final stage of our methodology, molecular docking, molecular dynamics simulation, and toxicity testing played a role in further exploring the complexity of different receptors interacting with PMMA-based materials to better understand their potential impact on oral and dental health. These receptors became more relevant to real-life clinical cases, providing a reliable theoretical solution for decision-making in dental material science—biomolecules involved in inflammation induced by PMMA-based dentures.

### 3D Structure modeling of MMA and PMMA with MM2 energy minimization

Minimizing the MMA energy led to a total energy of 11.2863 kcal/mol. The breakup of the energy component helps us comprehend all molecular dynamics. It is clear from Table 2 that the energies derived from stretching and bending are 0.8416 and 3.2643 kcal/mol, respectively. These energies represent the strain forces and elasticities among the bonds and the bond angles in the MMA molecular structure, respectively. The energy component of the torsion showed a rotational strain of −0.7204 kcal/mol. The non-1,4 and 1,4 VDW were 0.8763 and 6.1160 kcal/mol, respectively. The dipole-dipole interaction was 0.8550 kcal/mol. It is clear from the total energy that the MM2 energy-minimization process yielded a stable 3D structure for MMA, verifying the use of the initial geometry for subsequent simulations. In contrast, PMMA had a considerably high total energy of 133.5106 kcal/mol after its minimization. The individual energy components illustrate the significant molecular dynamics of PMMA. The overall stretch and bend energies were relatively high at 12.6514 and 46.5357 kcal/mol, respectively, whereas the torsion energy related to the rotational strain was relatively high at 19.1089 kcal/mol. The non-1,4 VDW interaction energy was relatively high at −11.0507 kcal/mol, which implies potential repulsion or unfavorable contact with another –CH_[61]− group. Compared to this, the 1,4 VDW energy (i.e., the energy arising from the interactions of the C atoms in the middle with methyl groups on the neighboring chains) was relatively high at 50.7950 kcal/mol, implying favorable van der Waals (VDW) interactions. The PMMA dipole-dipole interaction was 11.5710 kcal/mol. Its relatively high total energy is related to the increased strain in its molecular architecture compared to MMA’s relatively low-strain molecular architecture. The table also shows the partial energy associated with the depreciation of PMMA. The total energy of PMMA represented a 33.3598 kcal/mol increase from the energy of MMA after minimization. The complete minimization steps and results are presented in Supplementary Data 1.

**Table 2:** Comparison of energetic parameters between MMA and PMMA after MM2 energy minimization

Parameter (kcal/mol)	MMA	PMMA
Stretch	0.8416	12.6514
Bend	3.2643	46.5357
Stretch-Bend	0.0535	3.8993
Torsion	−0.7204	19.1089
Non-1,4 VDW	0.8763	−11.0507
1,4 VDW	6.1160	50.7950
Dipole/Dipole	0.8550	11.5710
Total Energy	11.2863	133.5106

### Analyzing energy differences and molecular structures: implications for molecular docking and dynamics simulation

**Fig. 1:**
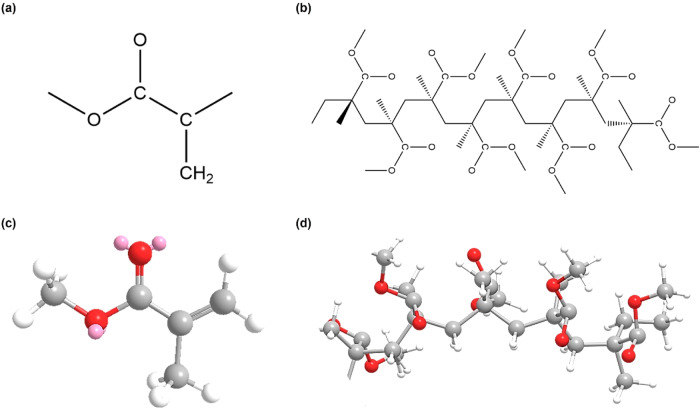
. Chemical visualization. **a** 2D structure of MMA. **b** 2D structure of PMMA. **c** Minimized 3D structure of MMA. **d** Minimized 3D structure of PMMA

The difference in the total energy between MMA and PMMA represents the energy implied by the two plastics’ different chemical compositions and molecular structures. MMA, being a monomeric unit, represents a smaller molecular structure with a lower total energy, which indicates the simple and more stable and relaxed 3D structure of MMA to PMMA with a higher Total Energy induced to its molecular structure. The molecular structure of PMMA as a polymer indicates lower stability and strain-absorbing quality owing to its larger molecular structure. This also leads to charged attraction from unpaired electrons with higher energy levels in the molecular structure, resulting in molecular oscillations. Therefore, the energy profiles from MM2 energy minimization are one of the key inputs required in other computational analyses, such as molecular docking and molecular dynamics simulations. The smaller molecular size of MMA makes it a more reliable template for 3D structure docking with its biomolecular targets. In contrast, the higher energy suggested for the molecular structure of PMMA indicates the requirement for an extensive analysis of its interaction with receptors, considering the extra strain that its larger molecular structure might be induced when binding with the receptor. Figure 1 shows the minimized MMA and PMMA structures. The next stage of our study involved molecular docking simulation and molecular dynamics analysis to determine how these energy-minimized structures interact with receptors involved in selected bone metabolism.

### Molecular docking simulation

**Fig. 2:**
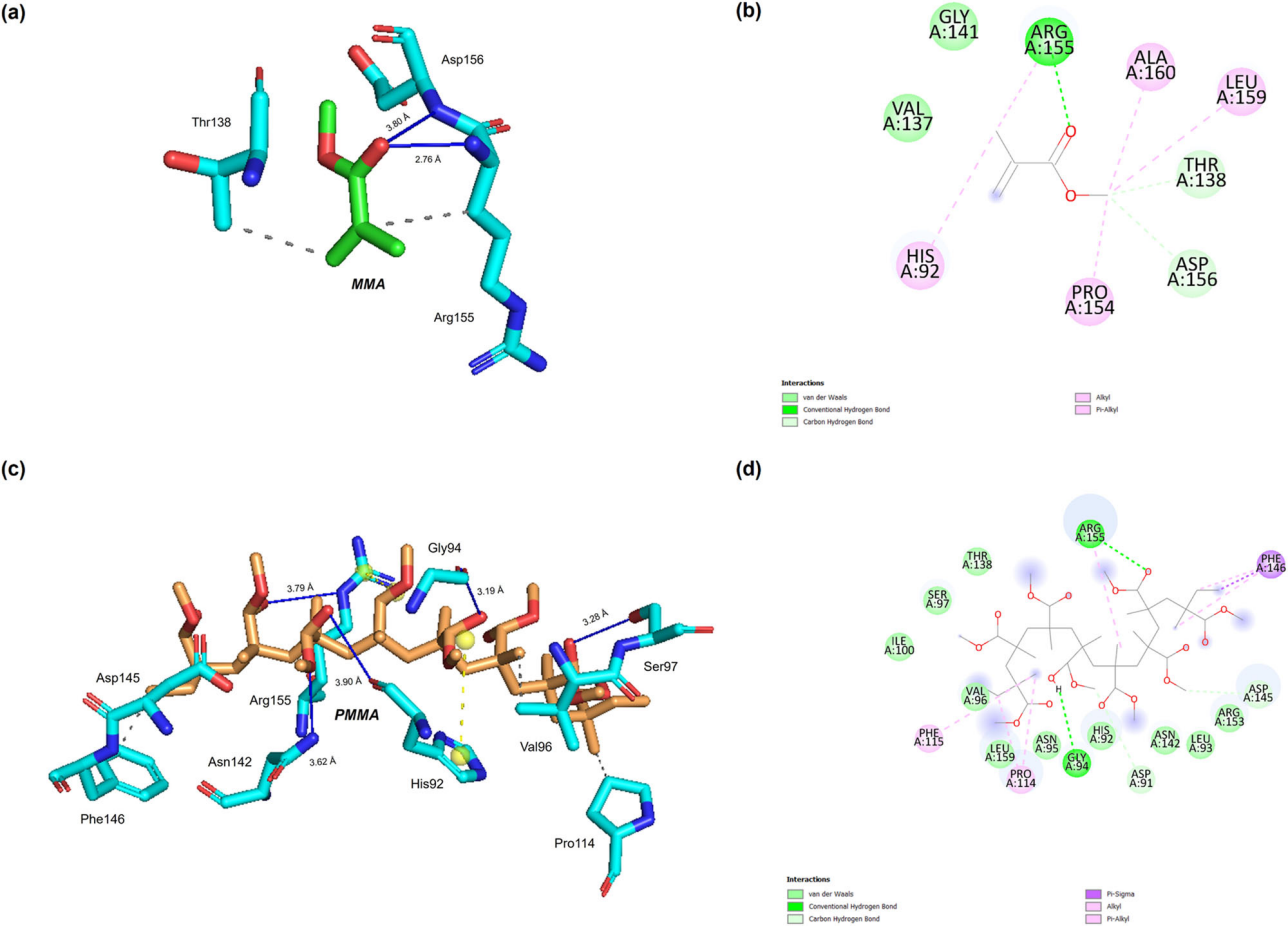
Molecular docking simulation results. **a** The 3D perspective of the TRAP: MMA complex. **b** The 2D perspective of the TRAP: MMA complex. **c** The 3D perspective of TRAP: PMMA complex. **d** The 2D perspective of TRAP: PMMA complex

Table 3 shows the molecular docking simulation results for the top five MMA complexes in quantitative and detailed forms, including the binding affinities and energetics and the interaction details with selected receptors. Molecular docking simulation is a comprehensive scientific endeavor that can offer micro-detailed insights into the biocompatibility of MMA for dental use, particularly in the realm of interim prosthetic restorations. The robust interactions observed in the RANKL: MMA and BMP9:MMA complexes were reflected in impressive HADDOCK scores of −14.3 ± 1.6 and −15.0 ± 0.1, respectively, suggesting that MMA may positively influence osteoclast differentiation and bone formation. The notable binding affinities, with binding energy ΔG values of −6.05 kcal/mol and −5.88 kcal/mol for RANKL: MMA and BMP9:MMA, respectively, underscore the strong affinity of MMA for these key receptors involved in bone metabolism. Similarly, the moderate interactions in TRAP: MMA and NOTCH2:MMA complexes, with HADDOCK scores of −4.9 ± 1.2 and −7.6 ± 2.9, respectively, indicate potential regulatory roles in bone remodeling and tissue development. The binding affinity values for TRAP: MMA and NOTCH2:MMA, with ΔG values of −5.92 kcal/mol and −5.88 kcal/mol, suggest favorable binding strengths, contributing to the diverse impacts of MMA on biomolecular targets. The strong interaction observed in the fibronectin-MMA complex (HADDOCK score of −10.7 ± 0.6) implies a potential role in supporting the extracellular matrix in bone tissue. The energetics and stability indices, together with the binding affinities observed for the five best MMA complexes, ultimately support the robustness of the proposed hypothesis by demonstrating the formation of MMA complexes with strong binding affinities for biocompatible biomolecules preferable for dental materials, especially interim prosthetic restorations. Molecular docking simulations showed that the top five PMMA complexes in the model represent the PMMA-based dental material with potential biocompatibility, with three of the most critical receptors in the regulation of key functions and pathways in dental tissue health. Particularly, the lowest energy PMMA-AP complex with a HADDOCK score of −36.4 ± 2.0 and a binding affinity of −9.36 kcal/mol indicates that PMMA might be one of the main agents in driving cell adherence and mediating extracellular matrix interactions that may play an important role in the performance and integrity of the interim restorations within an oral cavity.The substantial binding affinity of PMMA with TRAP, as evidenced by the TRAP: PMMA complex with a ΔG value of −9.20 kcal/mol and a HADDOCK score of −33.1 ± 2.0, suggests potential implications for bone resorption processes. This finding is particularly relevant in dental applications, where PMMA-based materials may influence bone remodeling dynamics and contribute to the overall stability of prosthetic restorations. The interactions observed in the BMP3:PMMA complex with a HADDOCK score of −32.6 ± 1.0 and a binding affinity of −8.58 kcal/mol highlight PMMA’s potential influence on bone morphogenetic protein signaling pathways. This is pertinent to dental biocompatibility, as these pathways play a crucial role in regulating bone formation and regeneration, thereby impacting the long-term success of dental prostheses. Moreover, the interactions of PMMA with Osteonectin and Osteoprotegerin, represented by HADDOCK scores of −29.5 ± 2.5 and −24.0 ± 4.6, respectively, and binding affinities of −8.50 kcal/mol, indicate potential contributions to the regulation of bone matrix and osteoclast differentiation. These findings suggest that PMMA-based dental materials may influence the intricate balance of bone metabolism, an essential aspect of biocompatibility in dental applications. PMMA’s significant interactions between PMMA and key receptors involved in bone metabolism and cellular processes essential to dental prostheses were shown by molecular docking simulations. These findings highlight the potential of PMMA dental biocompatibility to support interim prosthetic restorations that interact well in complex oral environments. The MD simulations have clarified the dynamic behavior of the complexes, improving our understanding of PMMA’s long-term effects of PMMA and optimizing PMMA-based dental materials for biocompatibility and patient well-being. The full docking simulation results are presented in Supplementary Data 2. Table 4 illustrates the docking simulation results for the top five PMMA complexes. Figure 2 shows molecular docking simulations, showing that the top five PMMA complexes in the model represent the PMMA-based dental material with potential biocompatibility, with three of the most critical receptors in regulating key functions and pathways in dental tissue health. Particularly, the lowest energy PMMA-AP complex with a HADDOCK score of −36.4 ± 2.0 and a binding affinity of −9.36 kcal/mol indicates that PMMA might be one of the main agents in driving cell adherence and mediating extracellular matrix interactions that may play an important role in the performance and integrity of the interim restorations within an oral cavity.In the TRAP: MMA complex, the interaction was characterized by one conventional hydrogen bond and four carbon-hydrogen bonds. This specific binding pattern indicates a nuanced molecular interaction, where MMA forms stable connections with TRAP, potentially influencing cellular responses and bone metabolism. The presence of conventional hydrogen bonds underscores the specificity of the interaction, emphasizing the role of key molecular forces in mediating the biocompatibility of MMA with TRAP. The TRAP: PMMA complex exhibited a more intricate interaction profile featuring two conventional and impressive 12-carbon hydrogen bonds. This increased number of bonds suggests a stronger and more extensive binding affinity between PMMA and TRAP. The diverse nature of the bonds formed highlights the versatility of PMMA in establishing robust connections with TRAP, potentially impacting the regulation of osteoclast differentiation and bone remodeling processes. Many carbon-hydrogen bonds in this complex emphasize the hydrophobic contact between PMMA and TRAP, making it stable. These findings emphasize the role of TRAP in the PMMA biological receptor interactions between MMA and PMMA. TRAP’s role in bone resorption and remodeling makes its interaction with dental materials important for understanding osseointegration and biocompatibility. The interactions are explained at the molecular level in Fig. 2, and the particular bond counts establish the groundwork for molecular dynamics simulations. Supplementary Data 3 contains all MMP and PMMA complexes.

**Table 3:** Molecular docking simulation results for the top 5 MMA complexes

Complex	HADDOCK Score	Binding affinity ΔG (kcal/mol)	Ki (µM)	Cluster size	RMSD	Van der Waals energy	Electrostatic energy	Desolvation energy	Restraints violation energy	Buried surface area	Z-score
RANKL: MMA	−14.3 ± 1.6	−6.05	2.65 × 10^−5^	6	0.1 ± 0.1	−10.3 ± 1.1	−24.3 ± 7.9	−1.5 ± 0.2	0.1 ± 0.0	303.7 ± 11.5	−2.2
TRAP:MMA	−4.9 ± 1.2	−5.92	2.07 × 10^−5^	32	0.1 ± 0.1	−8.6 ± 1.4	−11.1 ± 1.1	−2.9 ± 0.5	76.1 ± 22.7	289.3 ± 3.9	−2.4
Fibronectin:MMA	−10.7 ± 0.6	−5.90	3.34 × 10^−5^	24	0.1 ± 0.1	−8.7 ± 0.8	−21.3 ± 4.9	−0.1 ± 1.0	2.0 ± 1.4	263.3 ± 3.1	−1.6
BMP9:MMA	−15.0 ± 0.1	−5.88	3.71 × 10^−5^	67	0.1 ± 0.1	−9.3 ± 0.2	−14.6 ± 1.9	−4.2 ± 0.3	0.1 ± 0.1	270.3 ± 5.5	−2.0
NOTCH2:MMA	−7.6 ± 2.9	−5.88	3.71 × 10^−5^	11	0.4 ± 0.0	−7.5 ± 0.9	−17.8 ± 2.3	−2.6 ± 0.2	43.5 ± 26.6	312.1 ± 3.6	−1.8

**Table 4:** Molecular docking simulation results for the top 5 PMMA complexes

Complex	HADDOCK Score	Binding affinity ΔG (kcal/mol)	Ki (µM)	Cluster size	RMSD	Van der Waals energy	Electrostatic energy	Desolvation energy	Restraints violation energy	Buried surface area	Z-score
AP:PMMA	−36.4 ± 2.0	−9.36	1.67 × 10 − 8	4	0.4 ± 0.0	−29.3 ± 2.1	3.5 ± 4.2	−9.2 ± 0.5	16.9 ± 7.9	1063.4 ± 24.5	−2.2
TRAP:PMMA	−33.1 ± 2.0	−9.20	3.89 × 10 − 9	23	0.1 ± 0.1	−29.2 ± 3.0	11.7 ± 3.0	−7.9 ± 1.8	28.4 ± 16.2	973.7 ± 27.4	−1.9
BMP3:PMMA	−32.6 ± 1.0	−8.58	1.67 × 10 − 7	22	0.4 ± 0.0	−33.0 ± 1.9	−14.8 ± 4.1	−0.7 ± 0.5	24.8 ± 23.8	808.2 ± 33.9	−1.9
Osteonectin:PMMA	−29.5 ± 2.5	−8.50	8.99 × 10 − 8	18	0.2 ± 0.1	−25.4 ± 2.1	−20.2 ± 1.1	−4.7 ± 0.3	26.8 ± 24.8	771.5 ± 30.4	−1.1
Osteoprotegerin:PMMA	−24.0 ± 4.6	−8.50	8.99 × 10 − 8	6	0.2 ± 0.2	−26.8 ± 1.8	3.0 ± 4.6	−4.2 ± 0.6	66.5 ± 30.8	754.4 ± 12.5	−1.6

### Pharmacophore modeling to assess active functional groups of MMA and PMMA

Table 5 shows that pharmacophore modeling was instantly applied to the retrospective analysis of the active functional groups engaged in the interaction of MMA and PMMA with the chosen receptors. The 2D and 3D structure-based pharmacophore models provided valuable insights into the nature of the molecular interactions at the binding sites of the receptors. In MMA complexes, the analysis revealed that carbonyl groups play a predominant role by forming hydrogen bonds as acceptors within the selected receptors. This underscores the significance of these specific functional groups in mediating the interaction between MMA and receptors.

**Table 5:** Based on the optimal docking conformation, pharmacophore models were constructed using 2D and 3D structural information

Complex	2D Pharmacophore	3D Pharmacophore
MMA Complexes		
RANKL: MMA		
TRAP: MMA		
Fibronectin: MMA		
BMP9:MMA		
NOTCH2:MMA		
PMMA Complexes		
AP: PMMA		
TRAP: PMMA		
BMP3:PMMA		
Osteonectin: PMMA		
Osteoprotegerin:PMMA		

In this representation, hydrophobic interactions, hydrogen bond donors, and hydrogen bond acceptors are depicted by yellow spheres, green arrows, and red arrows (spheres), respectively

The methyl group of MMA was observed to be involved mainly in hydrophobic interactions in the active sites of the receptors, thereby affecting complex stability and possibly giving overall predominance to these interactions, thereby contributing to the better binding affinity and biocompatibility of MMA with the selected receptors. The complete minimization steps and results are presented in Supplementary Data 4.

### Molecular dynamics (MD) simulation

**Fig. 3:**
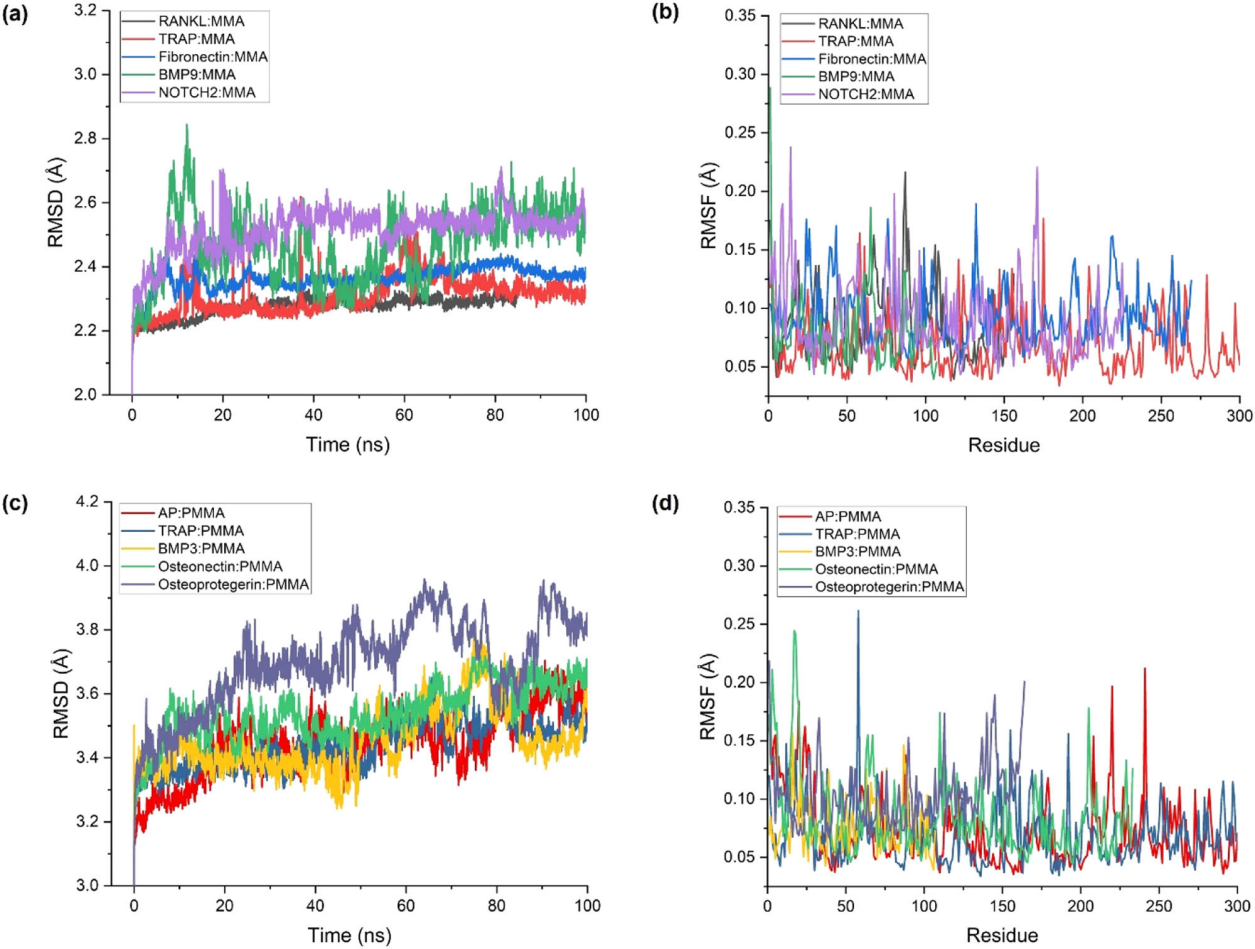
Molecular dynamics (MD) simulation results. **a** RMSD of top 5 MMA complexes. **b** RMSF of top 5 MMA complexes. **c** RMSD of top 5 PMMA complexes. **d** RMSF of top 5 PMMA complexes

MD simulations provide a deeper layer of understanding, and Fig. 3. offers a visual representation of the key parameters. Table 6 consolidates the time-averaged structural properties, enriching our insights into the dynamic behavior of the MMA and PMMA complexes. In-depth analysis of the MMA complexes provided valuable insights into their structural dynamics, emphasizing the robustness and stability of the key complexes. The average Root Mean Square Deviation (RMSD) values, ranging from 2.275 to 2.515 Å, underscore the consistent structural stability observed in the RANKL: MMA, TRAP: MMA, Fibronectin: MMA, BMP9:MMA, and NOTCH2:MMA complexes. These deviations represent the minimal structural fluctuations exhibited by the complexes during the MD simulations. Such steadiness is paramount in dental prosthetic materials, ensuring their resilience and structural integrity over time. Moreover, the examination of the Average Root Mean Square Fluctuation (RMSF) values, reflective of the flexibility of the complexes, and Average Radius of Gyration (RoG) values, indicative of their compactness, provided additional layers of understanding regarding the dynamic behavior of these materials. The low RMSF values suggest that specific residues within these complexes exhibit limited flexibility, contributing to their overall stability. Simultaneously, compactness, as indicated by the RoG values, reinforced the efficient packing and organization of the complexes, further supporting their suitability for dental applications. At the same time, the RMSD value of their structural stability was similar to that of MMA complexes, as described above. The RMSD values of the AP: PMMA, TRAP: PMMA, BMP3:PMMA, Osteonectin: PMMA, and Osteoprotegerin: PMMA complexes ranged from approximately 3.120 Å to 3.159 Å. These findings demonstrate the resistance of PMMA to dynamic conditions, representing many current dental prosthetic materials. They also suggested that PMMA can withstand the stresses provided by simulated environmental conditions. Furthermore, insights into the inherent flexibility and compactness of these PMMA complexes were gleaned from the analysis of the RMSF and RoG values. The low RMSF values indicate the limited flexibility of specific residues within the complexes, contributing to the overall stability of the PMMA complexes. The RoG values highlight the complexes’ efficient packing and organization, reinforcing the PMMA’s suitability for dental prosthetics. These findings collectively contribute to a comprehensive understanding of PMMA’s structural behavior of PMMA under dynamic conditions, reinforcing its viability and durability as a material for dental applications. These values reflect the system’s total stability and the varying interactions between complex components. Moreover, monitoring the total number of hydrogen bonds formed over the simulation time step revealed the nature of the interatomic interactions. These parameters complement each other to paint a more realistic picture of the interactions between MMA and PMMA and their respective biomolecular targets in the oral microenvironment. Combining this result with earlier findings by molecular docking highlighting the primary binding events of MMA or PMMA with their corresponding receptors deciphers the biocompatibility and stability of dental materials. Extensive investigation of their dynamic behaviors is crucial to ensure that these materials can bind efficiently with biological structures and that they are stable over time, both important for optimizing dental materials and further development for use in clinical practice. The docking and MD simulation integrative approach promoted the material design of dental technologies and the study of possible applications in dentistry. The complete MD simulation results are presented in Supplementary Data 5.

**Table 6:** Time-averaged structural properties obtained from molecular dynamics (MD) simulations of MMA and PMMA complexes

Complex	Average RMSD (Å)	Average RMSF (Å)	Average RoG (nm)	Potential energy (kJ/mol)	Number of hydrogen bonds
MMA Complexes					
RANKL: MMA	2.275	0.084	1.697	−935,490.639	9
TRAP:MMA	2.314	0.068	1.899	−1,369,006.445	12
Fibronectin:MMA	2.365	0.095	3.267	−1,100,808.251	7
BMP9:MMA	2.470	0.082	1.912	−1,202,818.975	6
NOTCH2:MMA	2.515	0.089	1.853	−1,086,019.608	7
PMMA Complexes					
AP:PMMA	3.120	0.072	2.324	−1,635,501.000	17
TRAP:PMMA	3.114	0.071	1.893	−1,344,982.178	22
BMP3:PMMA	3.116	0.076	1.867	−926,883.224	14
Osteonectin: PMMA	3.135	0.085	2.036	−1,259,171.389	17
Osteoprotegerin:PMMA	3.159	0.105	2.924	−1,050,569.023	19

### In silico toxicity assessment of MMA and PMMA

In silico toxicity assessments of MMA and PMMA were performed to understand their possible biological effects. Table 7 provides an insight into the overall prediction parameters related to toxicity, where the cLogP values for MMA and PMMA were 0.443 and 5.322, respectively. The notable increase in cLogP for PMMA suggests a more lipophilic nature than that of MMA. Lipophilicity plays a crucial role in drug absorption and distribution, and the significant differences observed between MMA and PMMA may have implications for bioavailability and pharmacokinetics. The total surface area of PMMA (672.10 Å²) was much higher than MMA’s (91.63 Å²). Therefore, they can interact differently with biological systems and their components. This can lead to differences in biocompatibility and toxicity. The relative PSA value of MMA (0.252 Å²) was higher than that of PMMA (0.274 Å²); however, the absolute value of the PSA of PMMA (235.68) was much higher than that of MMA (29.46). These values illuminate the potential interactions between these materials in polar and nonpolar environments. The in silico toxicity assessment also predicted effects on the endpoints of mutagenicity, tumorigenicity, reproductive effects, and irritancy—all of which were ‘None’ for MMA and PMMA, suggesting a favorable profile for both of these materials regarding genotoxicity and reproductive toxicity effects, that are usually the primary concerns. The Shape Index values for MMA (0.714) and PMMA (0.355) suggested differences in the molecular shapes of the two compounds. Molecular flexibility, as indicated by the respective values of 0.513 for MMA and 0.939 for PMMA, suggests that PMMA is more flexible than MMA, potentially influencing its interactions with biological receptors. A more detailed investigation of the molecular complexity yields, in principle, very similar values for MMA (0.536) and PMMA (0.546), indicating that they both show approximately the same structural complexity. The broadly defined parameter indicates the molecular structure’s overall complexity, and the values’ comparability suggests they share similar characteristics. The solvent-accessible surface area (SASA) values provided insights into the exposure of the compounds to the surrounding environment. PMMA exhibits a substantially larger SASA (1420.455 Å²) than MMA (282.558 Å²), signifying greater surface accessibility for PMMA. This parameter is crucial for understanding the potential interactions between these materials and biological molecules. SASA’s hydrophobic and hydrophilic components (FOSA and FISA) exhibit substantial differences between MMA and PMMA. PMMA had higher values for both, indicating a more pronounced interaction with hydrophobic and hydrophilic environments. Quantitative prediction models for various properties, such as QPlogHERG, QPPCaco, QPlogBB, QPPMDCK, QPlogKp, and QPlogKhsa, provide additional insights into the potential permeability, distribution, and bioavailability of these compounds. The values obtained for PMMA generally exhibited more negative scores than those obtained for MMA, suggesting differences in their pharmacokinetic properties. According to the in silico toxicity profile, lipophilicity, surface area, molecular shape, flexibility, molecular complexity, and affinity towards hydrophilic and hydrophobic environments make a difference between MMA and PMMA. The absence of predicted mutagenicity, tumorigenicity, reproductive effects, and irritancy indicates the favorable safety profiles of MMA and PMMA. However, the observed differences in various parameters highlight the importance of considering these factors in the context of dental biocompatibility and potential applications.

**Table 7:** In silico toxicity results of MMA and PMMA

Parameter	MMA	PMMA
cLogP	0.443	5.322
cLogS	−0.849	−3.750
Total Surface Area	91.63	672.10
Polar Surface Area (PSA)	0.252	0.274
Relative PSA	29.46	235.68
Mutagenic	None	None
Tumorigenic	None	None
Reproductive Effective	None	None
Irritant	None	None
Shape Index	0.714	0.355
Molecular Flexibility	0.513	0.939
Molecular Complexity	0.536	0.546
Solvent Accessible Surface Area (SASA)	282.558	1420.455
Hydrophobic Component of SASA (FOSA)	250.752	1228.346
Hydrophilic Component of SASA (FISA)	31.806	192.109
QPlogHERG	−1.813	−6.910
QPPCaco	4946.346	149.324
QPlogBB	0.163	−4.720
QPPMDCK	2784.770	63.340
QPlogKp	−1.818	−1.220
QPlogKhsa	−0.819	−1.514

### Clinical implications

Understanding the clinical implications of our findings was crucial for their application in prosthodontics practice. By elucidating the molecular interactions and biocompatibility of PMMA-based dental materials, clinicians could make more informed decisions regarding their use in various clinical scenarios. For instance, the identification of specific functional groups through pharmacophore modeling provided insights into the molecular determinants of biocompatibility [62, 63], guiding clinicians in material selection for interim prosthetic restorations. Moreover, the results from molecular docking and dynamics simulations shed light on the stability and interactions of PMMA with biological receptors. Our study expanded on these findings by providing detailed insights into the structural stability of PMMA and its interactions with relevant receptors. Clinicians could utilize this information to anticipate the behavior of PMMA-based materials in the oral environment, thereby optimizing treatment outcomes and patient satisfaction. Additionally, the toxicity assessments conducted in this study offered valuable insights into the safety profile of PMMA. Our study built upon this research by incorporating in silico toxicity assessments to predict potential risks associated with PMMA-based materials. Clinicians could use these insights to mitigate potential risks and ensure patient safety during dental procedures. Overall, the clinical implications of our study extended to improved patient care and treatment outcomes in prosthodontic practice. By incorporating the findings into clinical decision-making processes, clinicians could enhance the biocompatibility, stability, and safety of PMMA-based dental materials, ultimately benefiting the oral health and well-being of their patients.

## Conclusion

This study has provided a comprehensive examination of the biocompatibility of PMMA, employing a range of sophisticated computational methodologies. Through energetic minimization analysis, molecular docking, dynamics simulations, pharmacophore modeling, and in silico toxicity studies, we have gained valuable insights into the behavior and safety profile of PMMA and its precursor MMA. The analysis revealed distinct molecular behaviors between PMMA and MMA, elucidating their stable structures and promising biocompatibility. Furthermore, pharmacophore modeling identified key functional groups involved in critical interactions, such as carbonyl, hydroxyl, and methyl groups, offering a deeper understanding of their role in biocompatibility. The in silico toxicity assessments provided reassuring results, predicting an excellent safety profile for both materials. Differences in lipophilicity and molecular shape were discerned, further enriching our understanding of their biocompatibility. These findings highlight the potential of computational approaches in guiding the design and optimization of dental prosthetic materials to enhance biocompatibility. However, while our study offers valuable insights, further experimental validation is necessary to confirm the accuracy of these outcomes. Clinicians and technicians involved in dental prosthetics can leverage the insights provided by this research to make informed decisions regarding the selection and utilization of PMMA-based materials. By considering factors such as structural stability, molecular interactions, and safety considerations, practitioners can contribute to the advancement of biocompatible dental materials, ultimately improving patient outcomes and satisfaction.

## Supplementary information

Supplementary Data 1

Supplementary Data 1

Supplementary Data 3

Supplementary Data 4

Supplementary Data 5

## Data Availability

The data presented in this study are available on request from the corresponding author.

## Abbreviations

**BMP**: Bone morphogenetic proteins
**BPA**: Bisphenol-A
**DMP-1**: Dentin matrix acidic phosphoprotein 1
**FISA**: Hydrophilic component of SASA
**FOSA**: Hydrophobic component of SASA
**GAFF2**: General Amber force field 2
**HADDOCK**: High ambiguity driven protein-protein docking
**IGF-1**: Insulin-like growth factor 1
**MD**: Molecular dynamics
**MMA**: methyl methacrylate
**PME**: Particle Mesh Ewald
**PMMA**: Polymethyl methacrylate
**PSA**: Polar surface area
**RANKL**: Receptor activator of nuclear factor kappa-B ligand
**RMSD**: Root mean square deviation
**RMSF**: Root mean square fluctuation
**RoG**: Radius of gyration
**SASA**: solvent-accessible surface area
**TGF-β1**: Transforming growth factor beta 1
**TRAP**: Tartrate-resistant acid phosphatase.
