# Supplementary material for: In silico assessment of biocompatibility and toxicity: molecular docking and dynamics simulation of PMMA-based dental materials for interim prosthetic restorations
Source: J Mater Sci Mater Med. 2024 Jun 4;35(1):28. doi: 10.1007/s10856-024-06799-7 (PMC11150300; doi:10.1007/s10856-024-06799-7)
Supplement: Supplementary file 4 — Supplementary Data 4 [file 10856_2024_6799_MOESM4_ESM.docx]

| **Complex** | **2D Pharmacophore** | **3D Pharmacophore** |
| --- | --- | --- |
| **MMA Complexes** | | |
| AP:MMA | 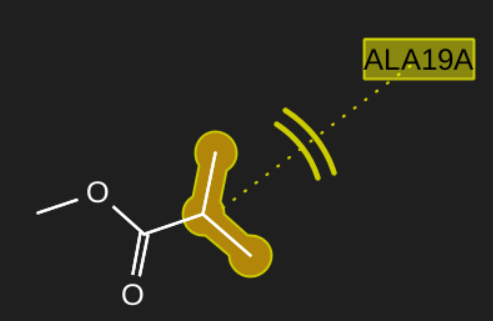 | 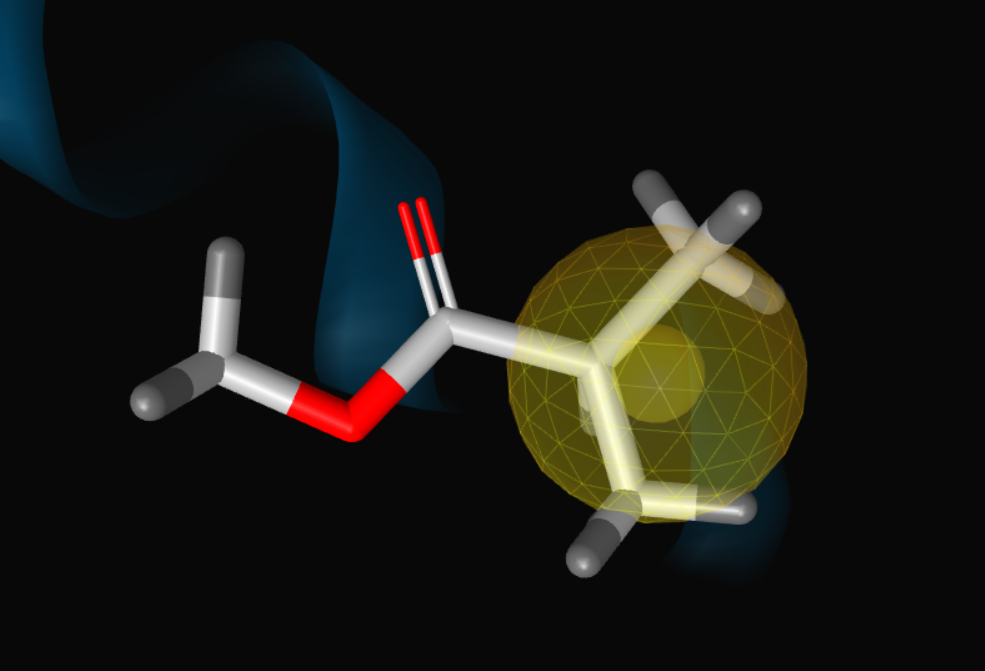 |
| BMP2:MMA | 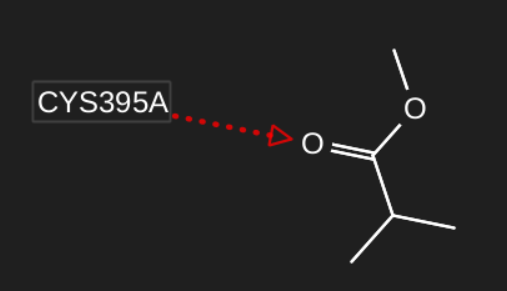 | 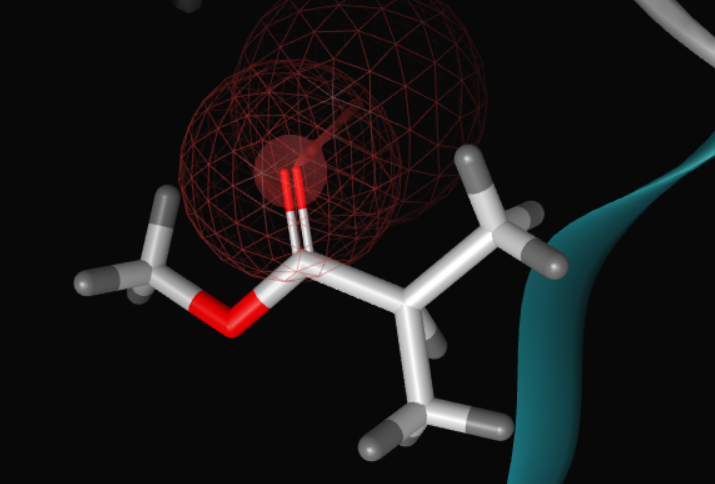 |
| BMP3:MMA | 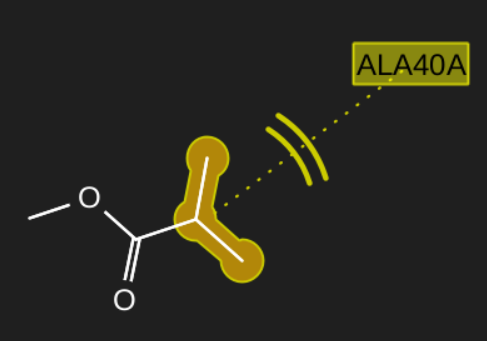 | 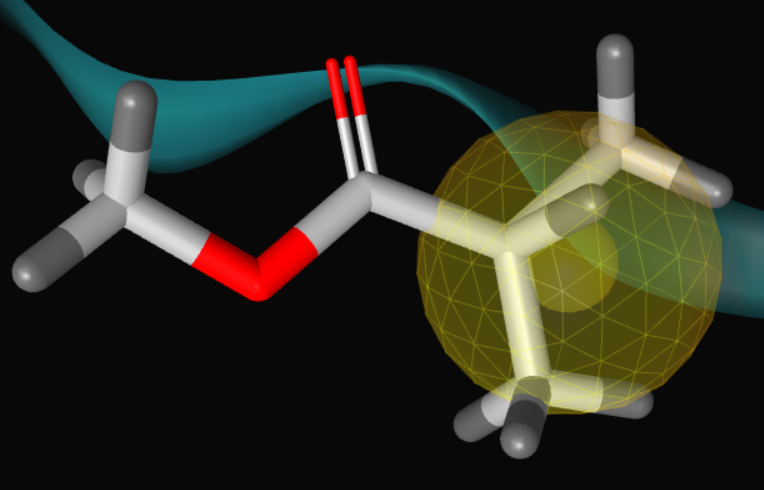 |
| BMP7:MMA | 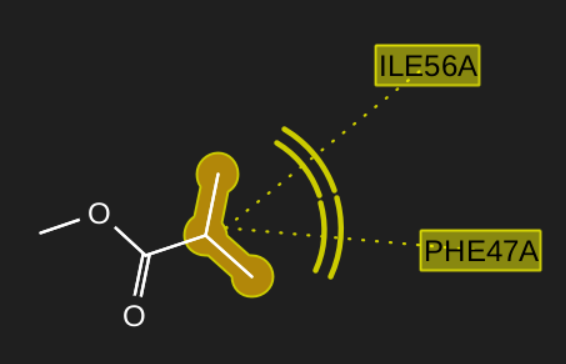 | 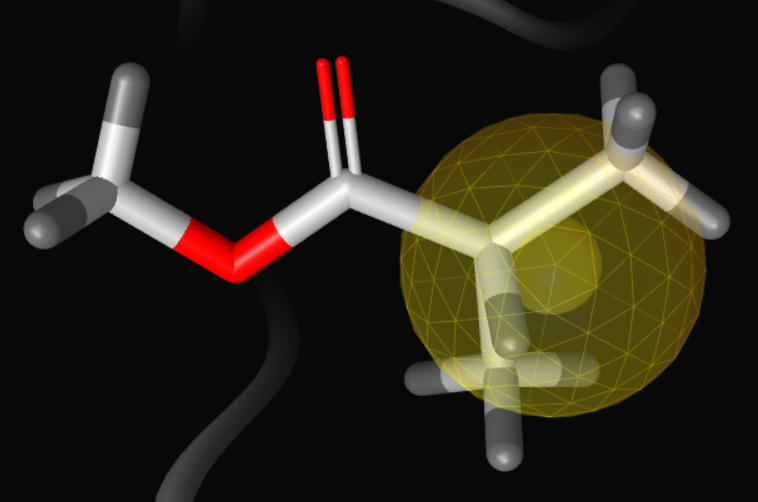. |
| BMP9:MMA | 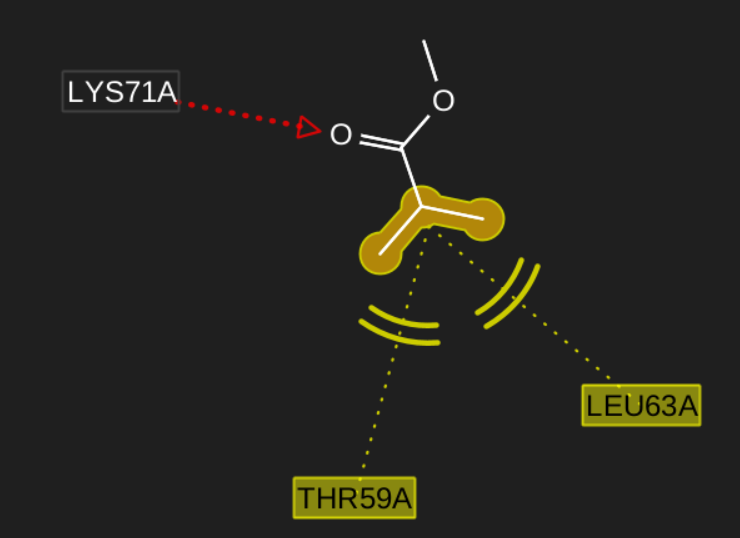 | 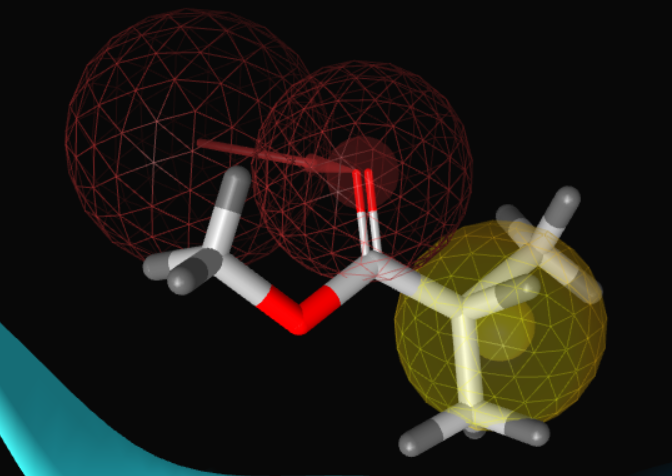 |
| COL1A1:MMA | 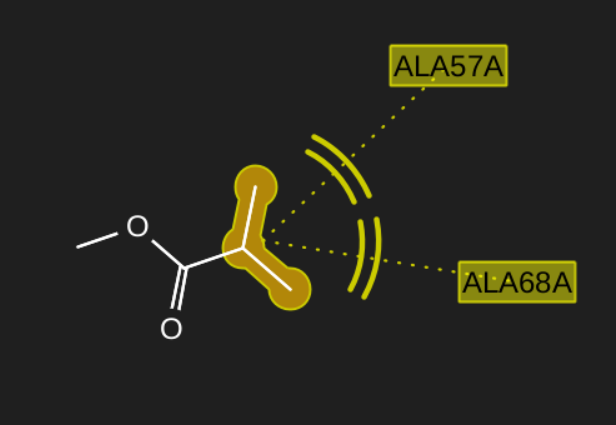 | 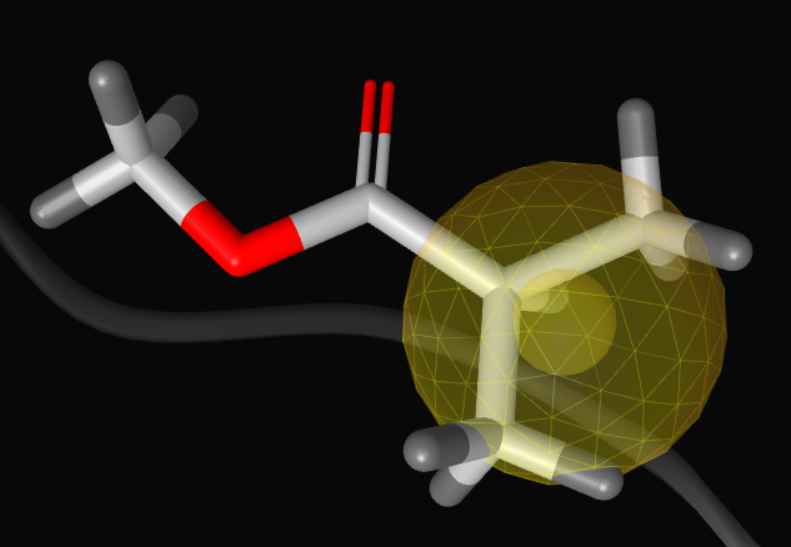 |
| DMP-1:MMA | 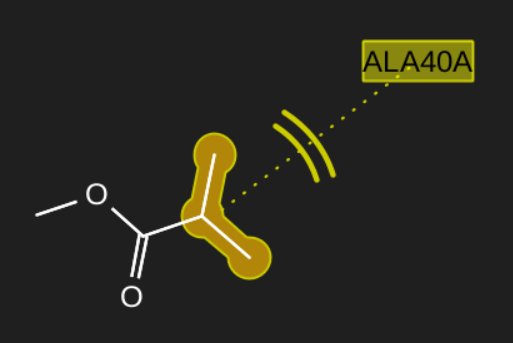 | 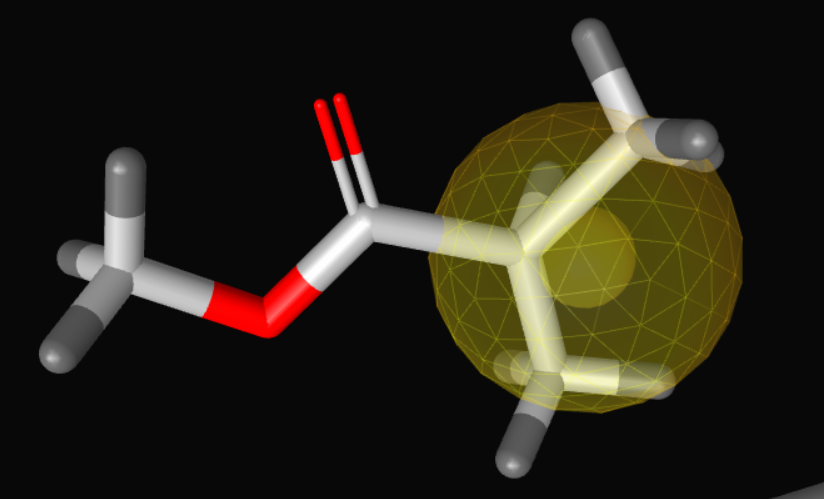 |
| Fibronectin:MMA | 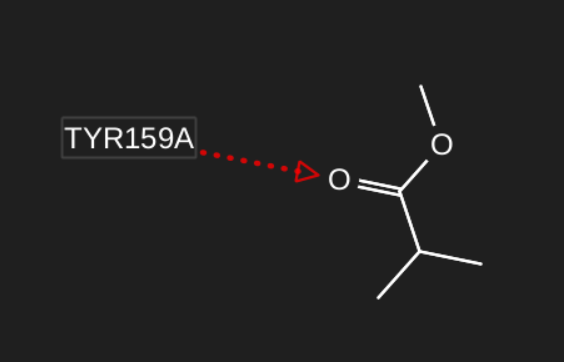 | 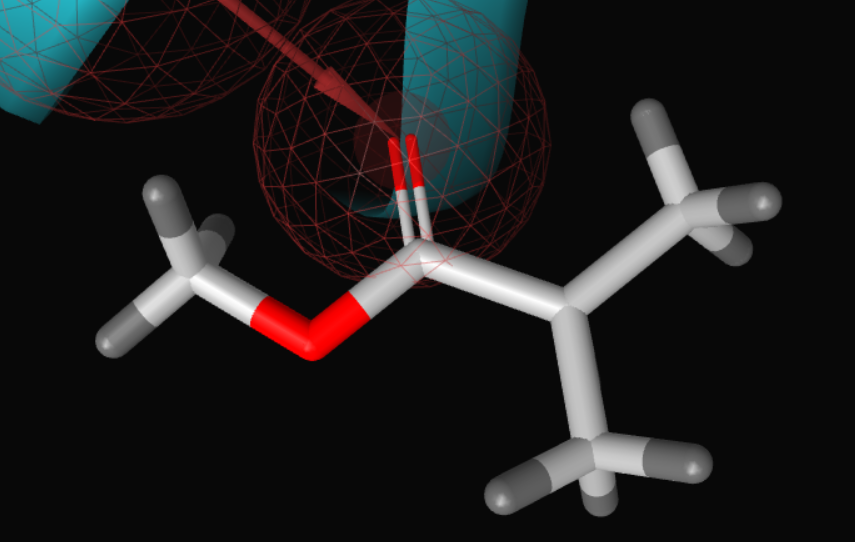 |
| IGF-1:MMA | 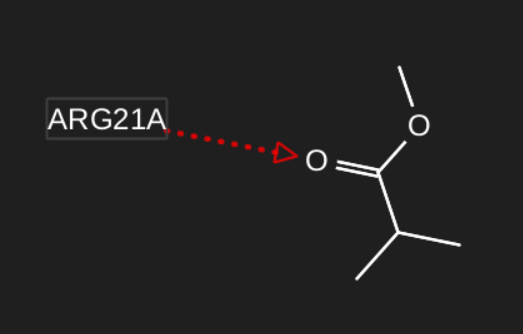 | 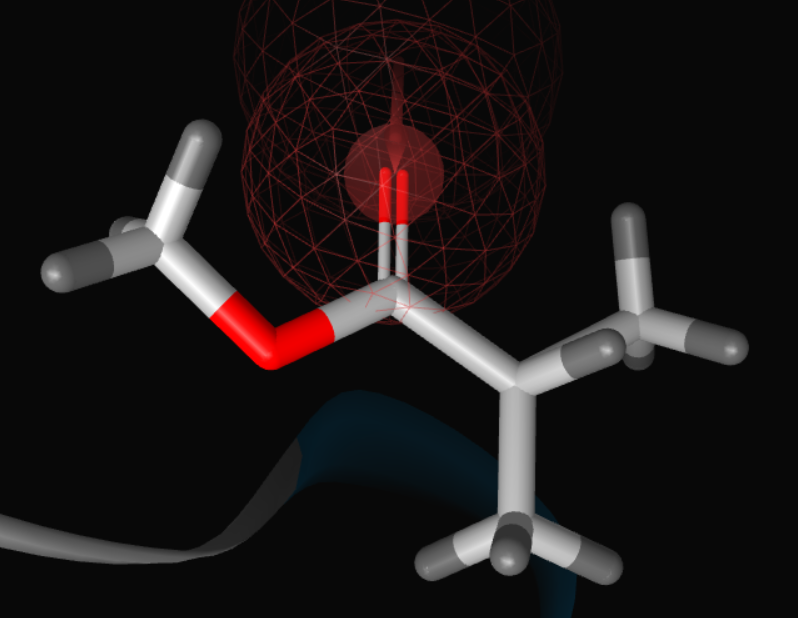 |
| NOTCH2:MMA | 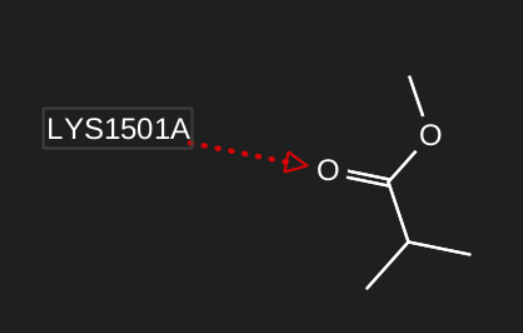 | 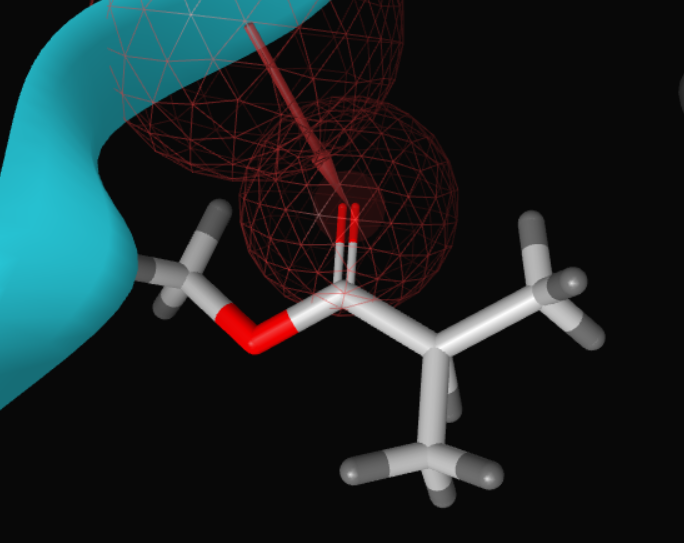 |
| Osteocalcin:MMA | 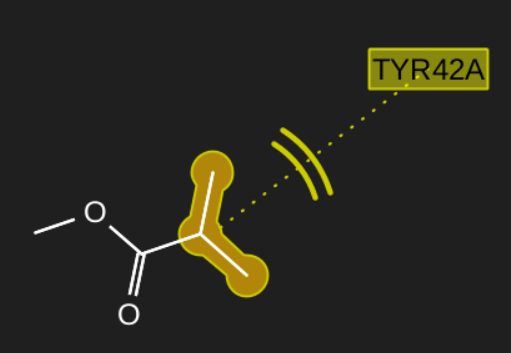 | 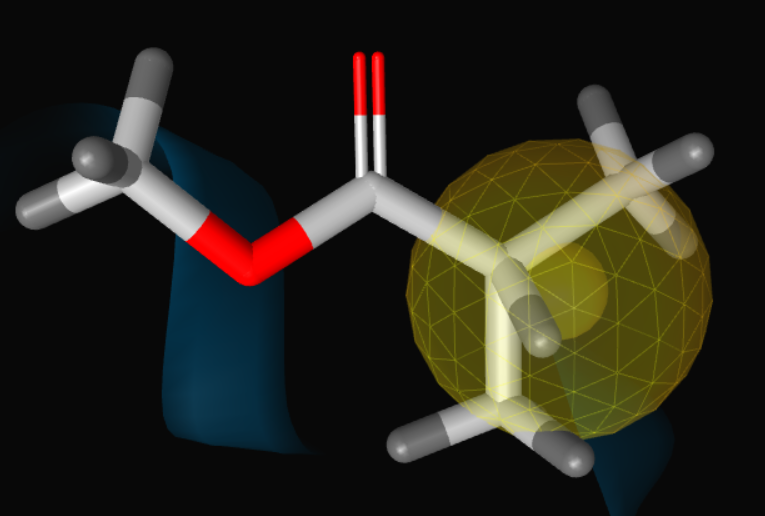 |
| Osteonectin:MMA | 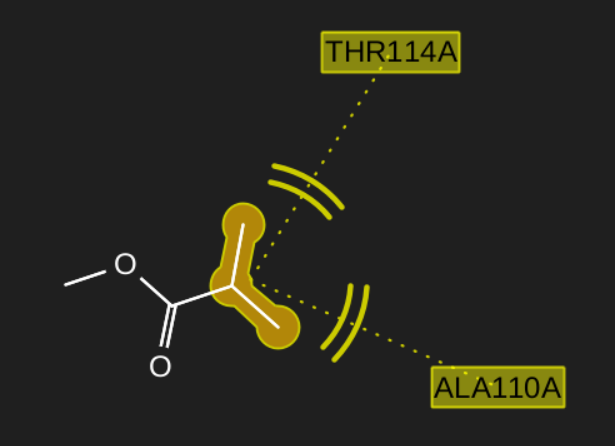 | 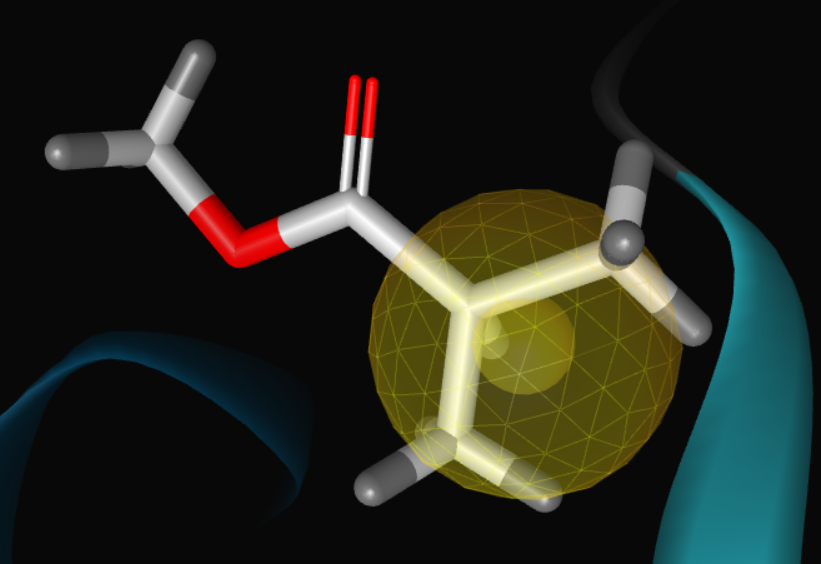 |
| Osteopontin:MMA | 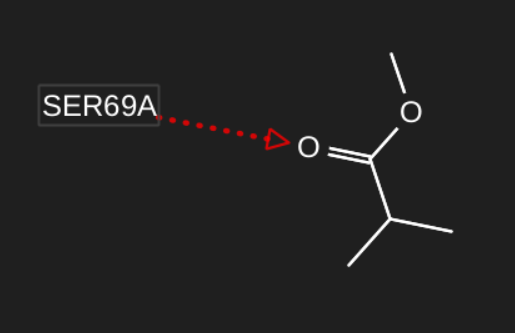 | 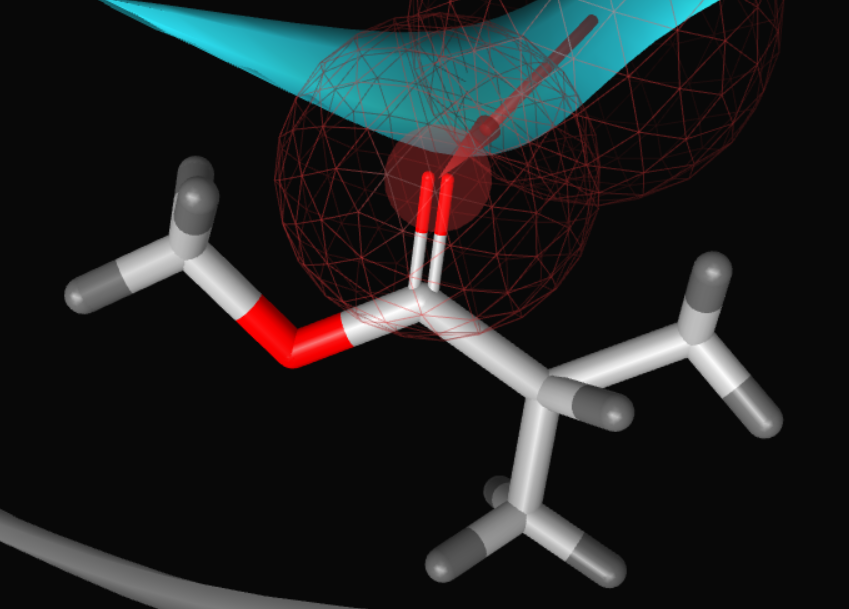 |
| Osteoprotegerin:MMA | 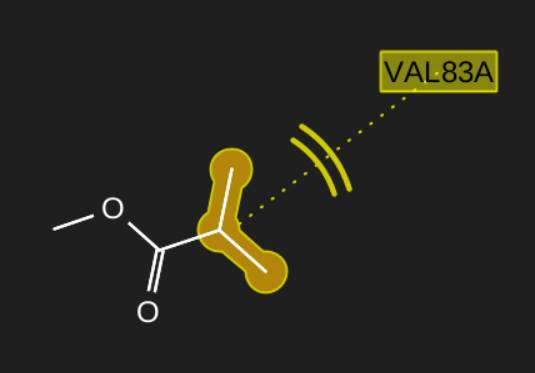 | 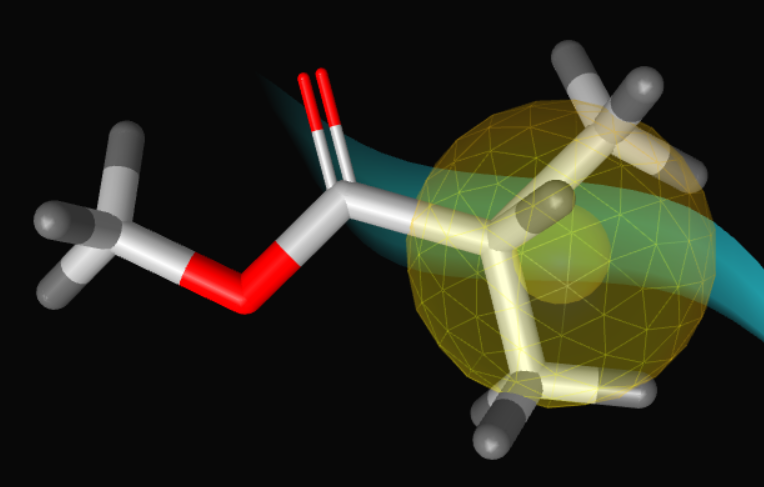 |
| Osterix:MMA | 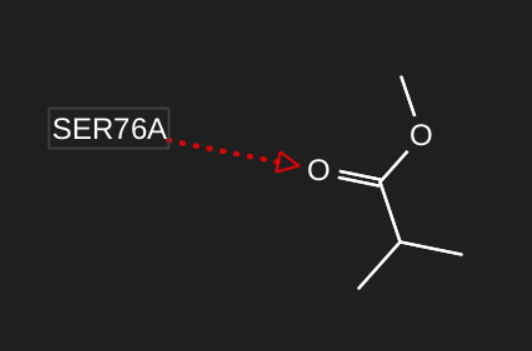 | 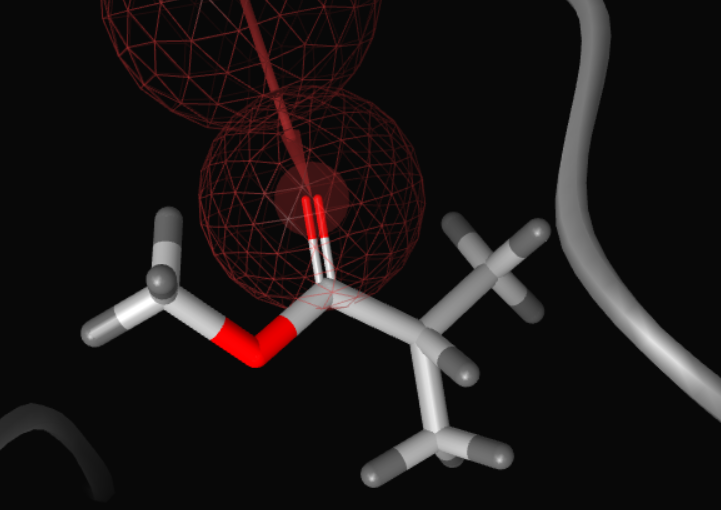 |
| RANKL:MMA | 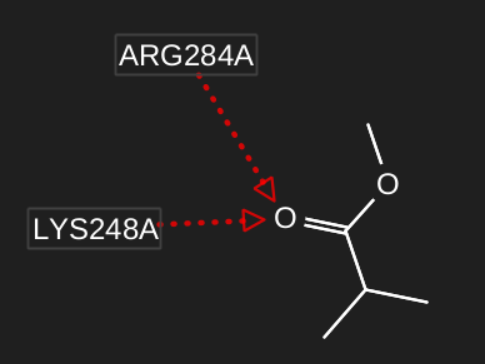 | 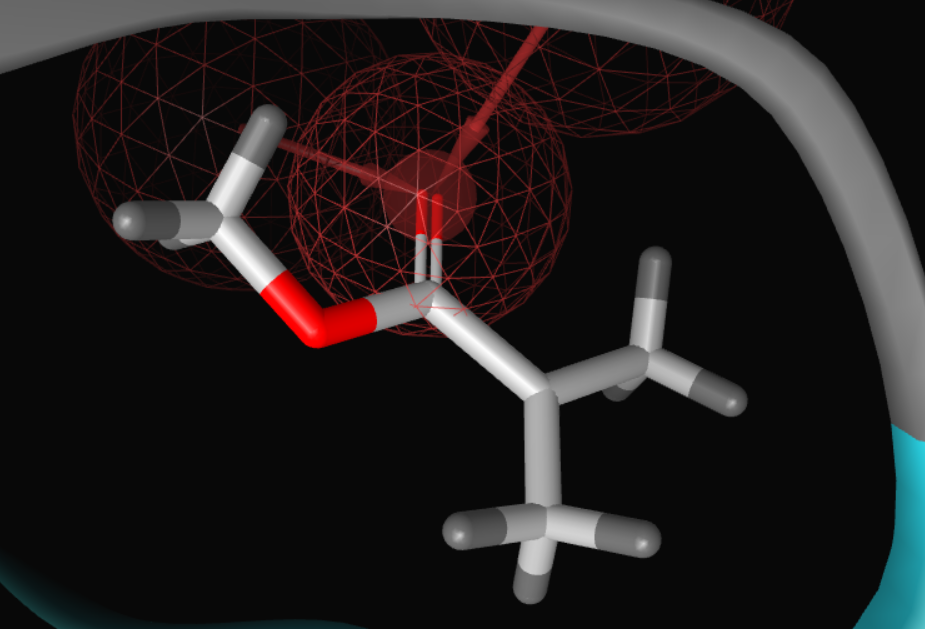 |
| RUNX2:MMA | 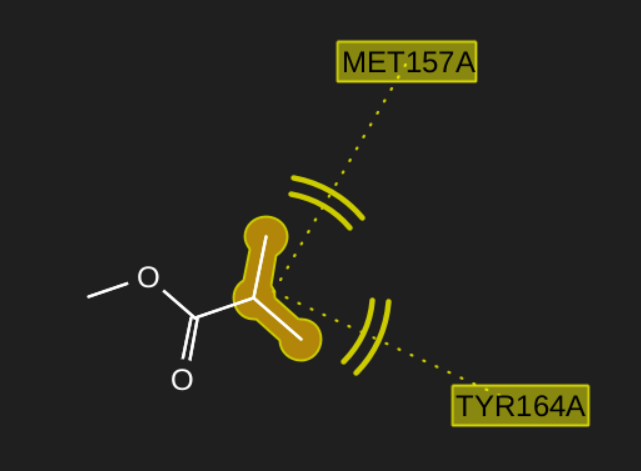 | 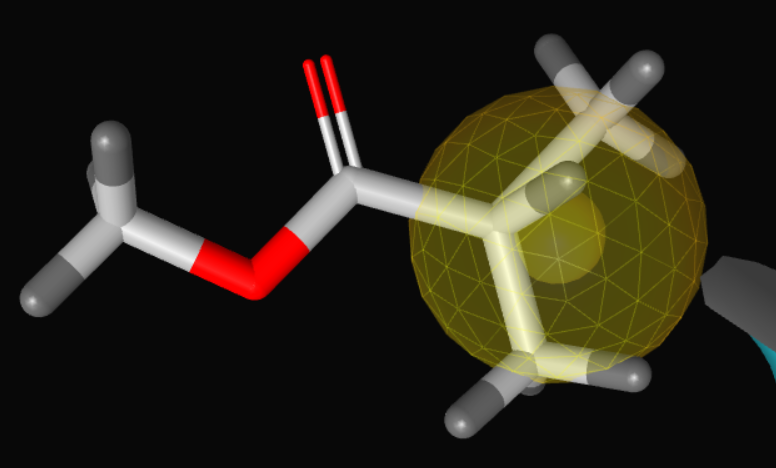 |
| TGF-B1:MMA | 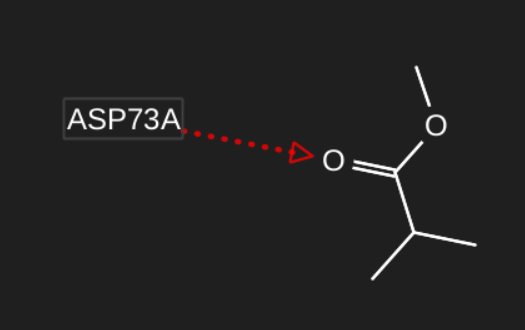 | 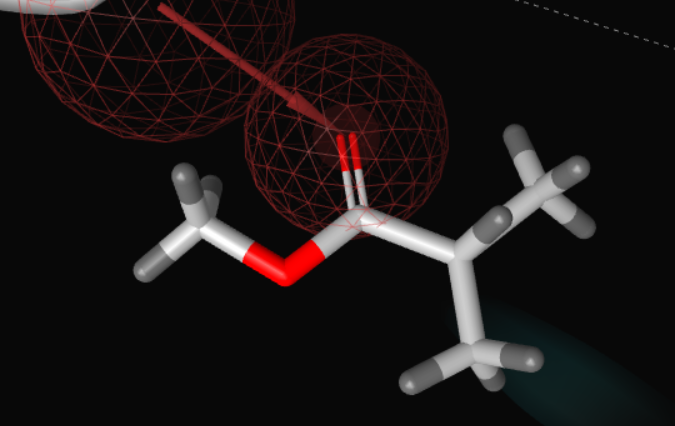 |
| TRAP:MMA | 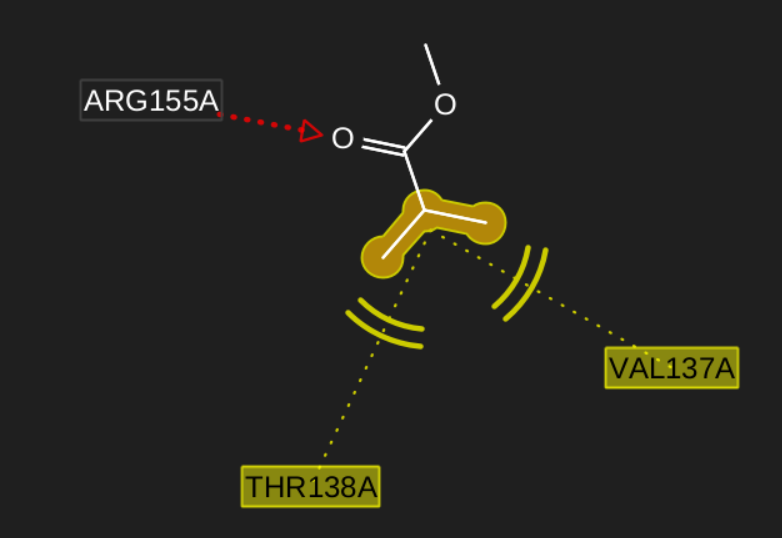 | 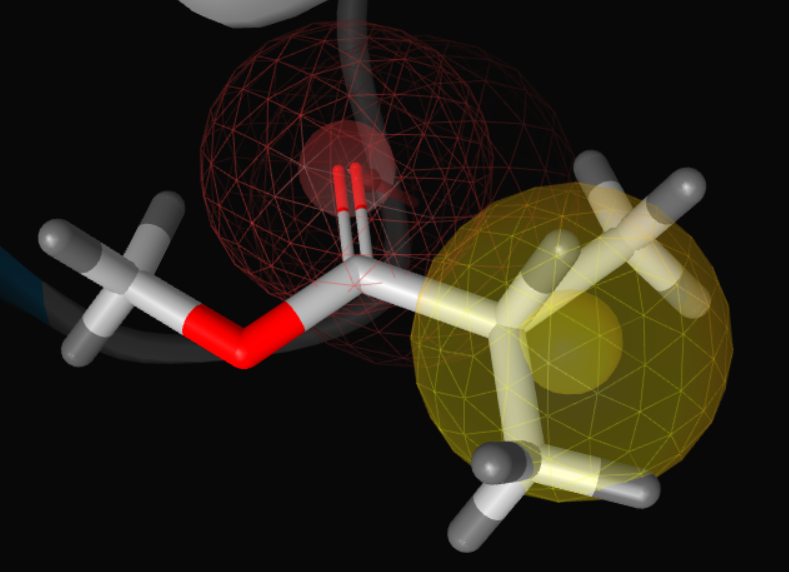 |
| Wnt3:MMA | 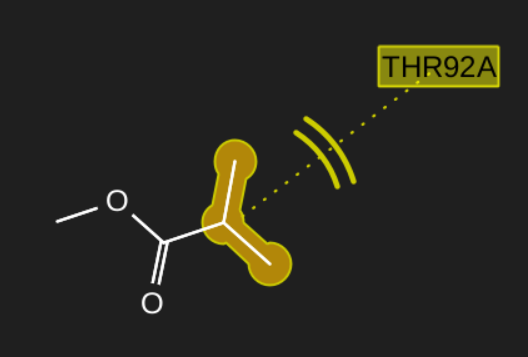 | 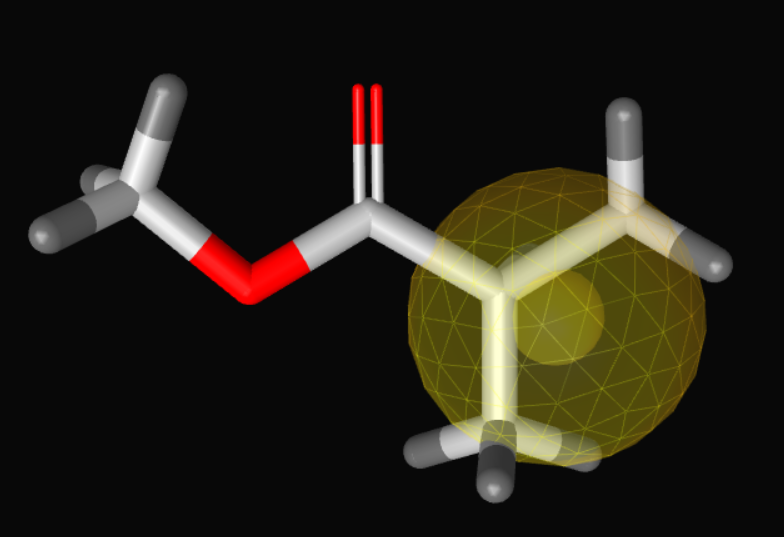 |
| **PMMA Complexes** | | |
| AP:PMMA | 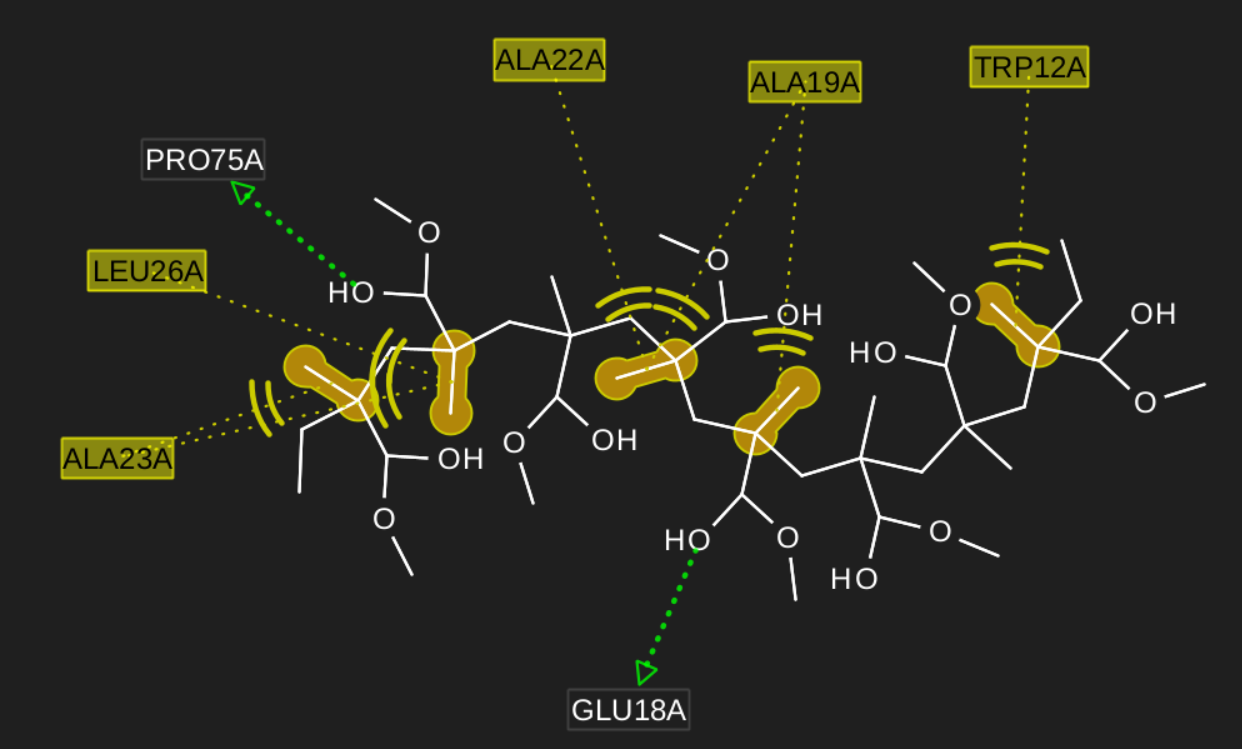 | 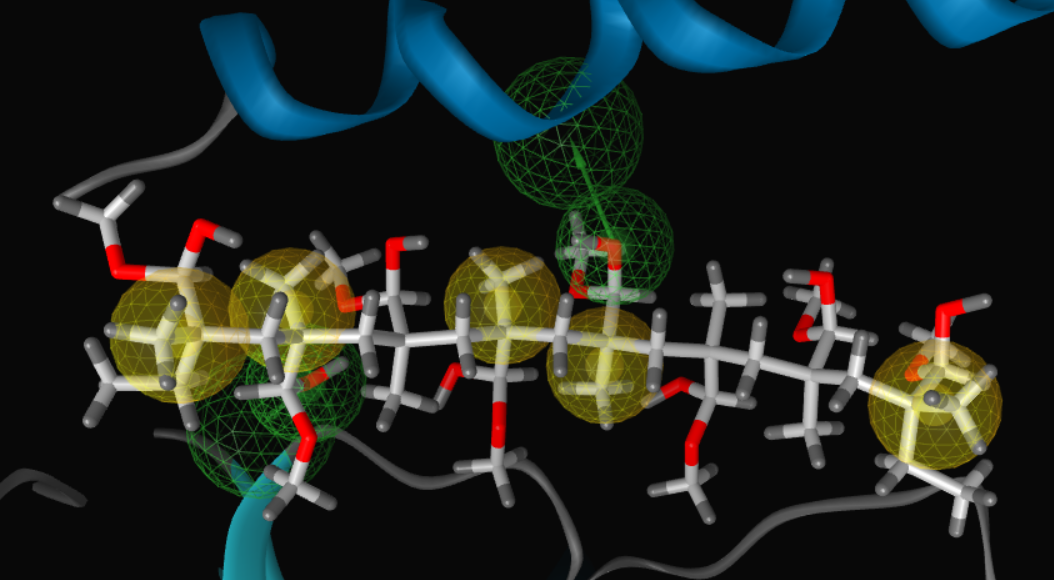 |
| BMP2:PMMA | 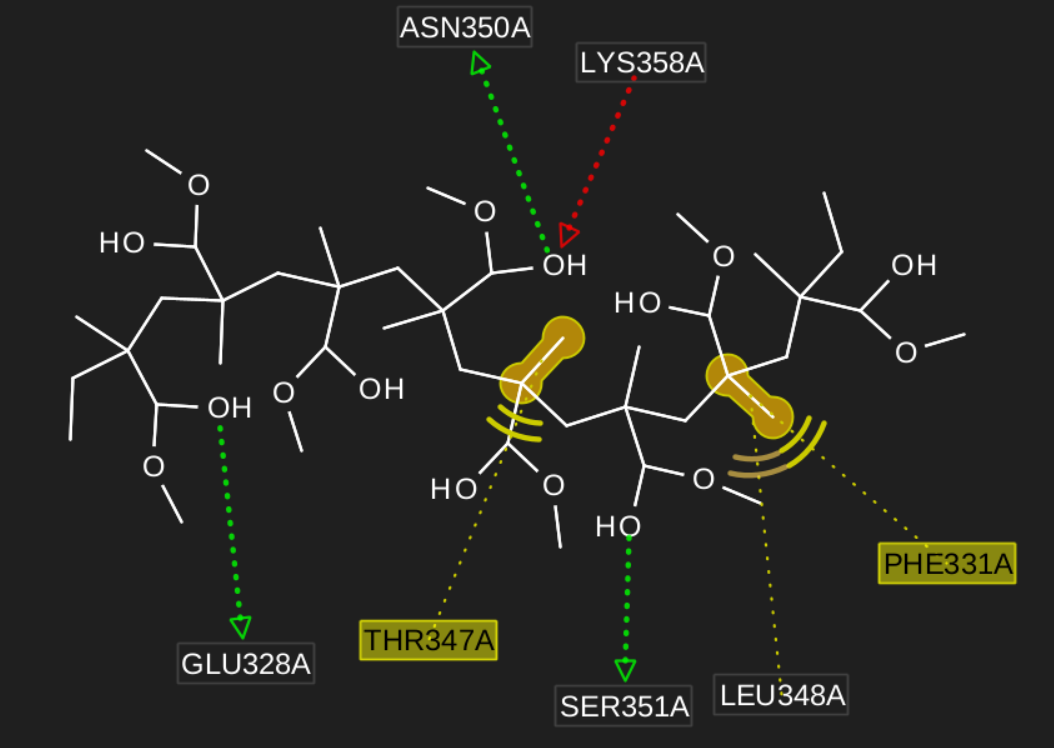 | 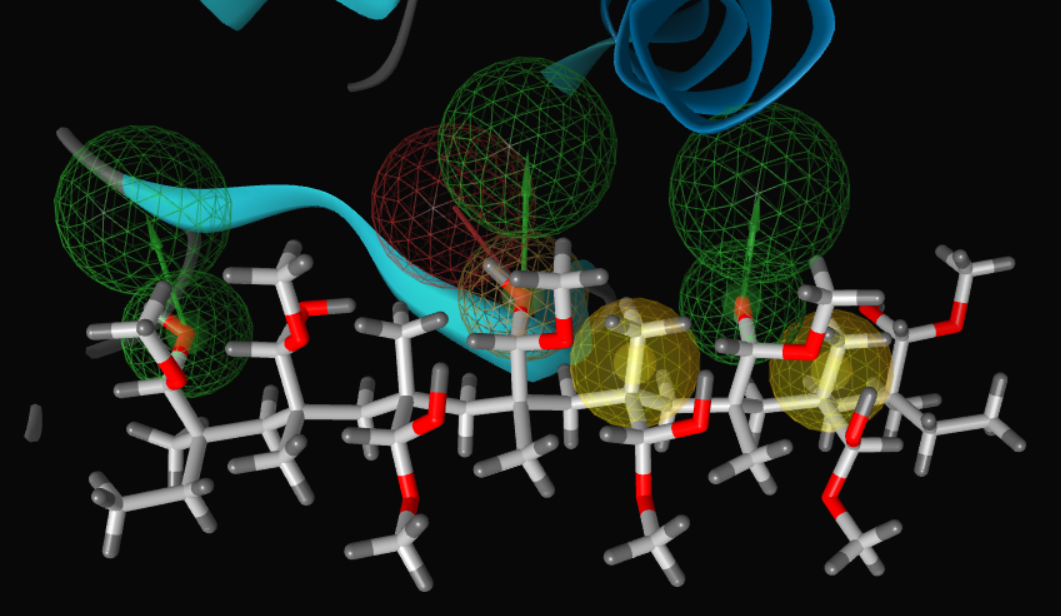 |
| BMP3:PMMA | 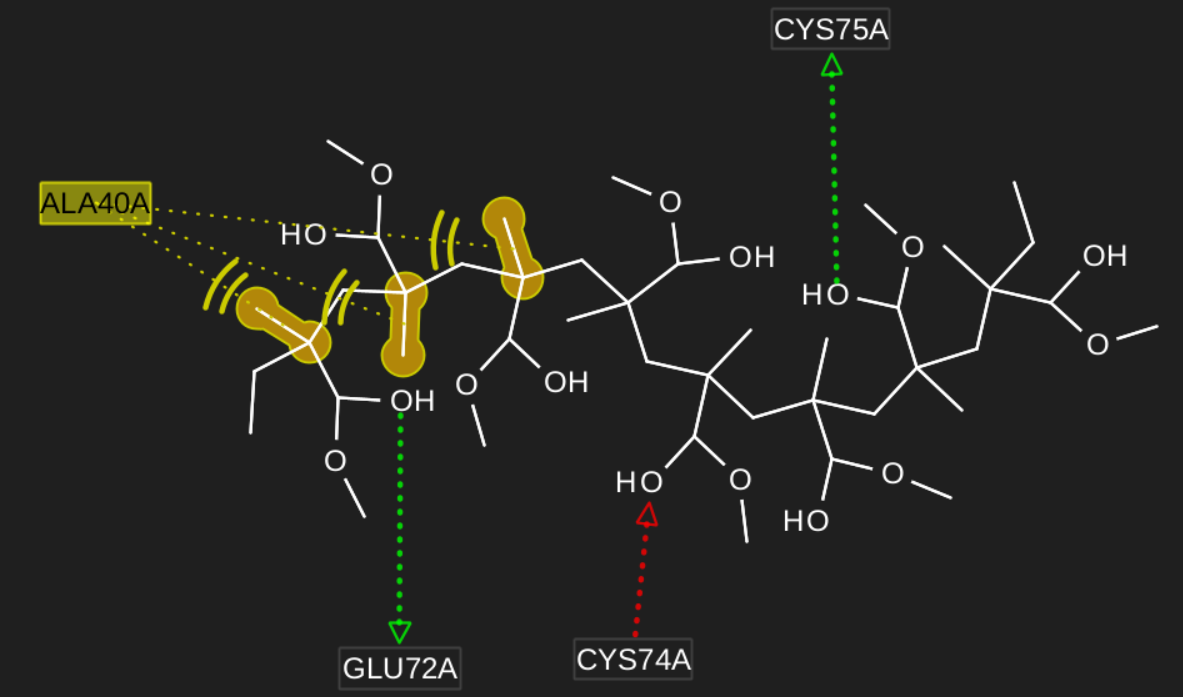 | 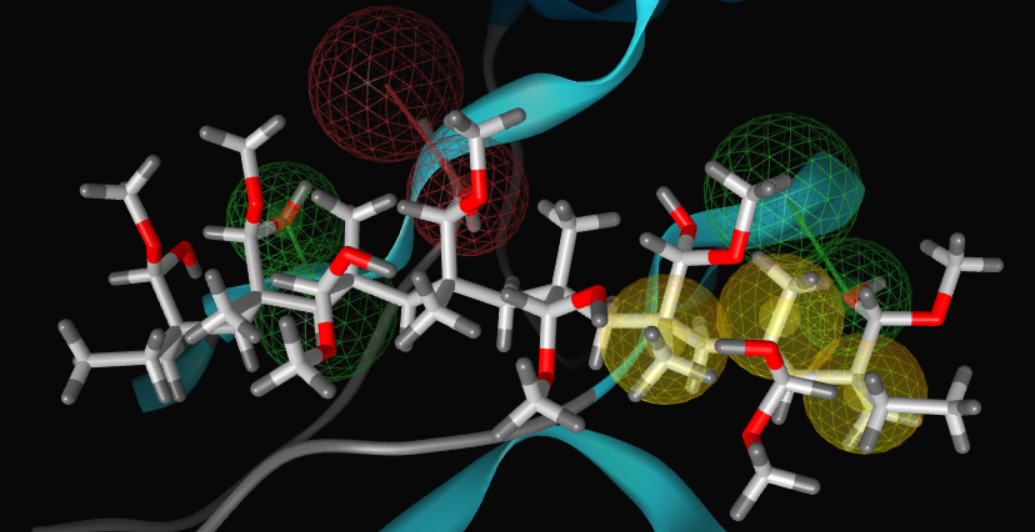 |
| BMP7:PMMA | 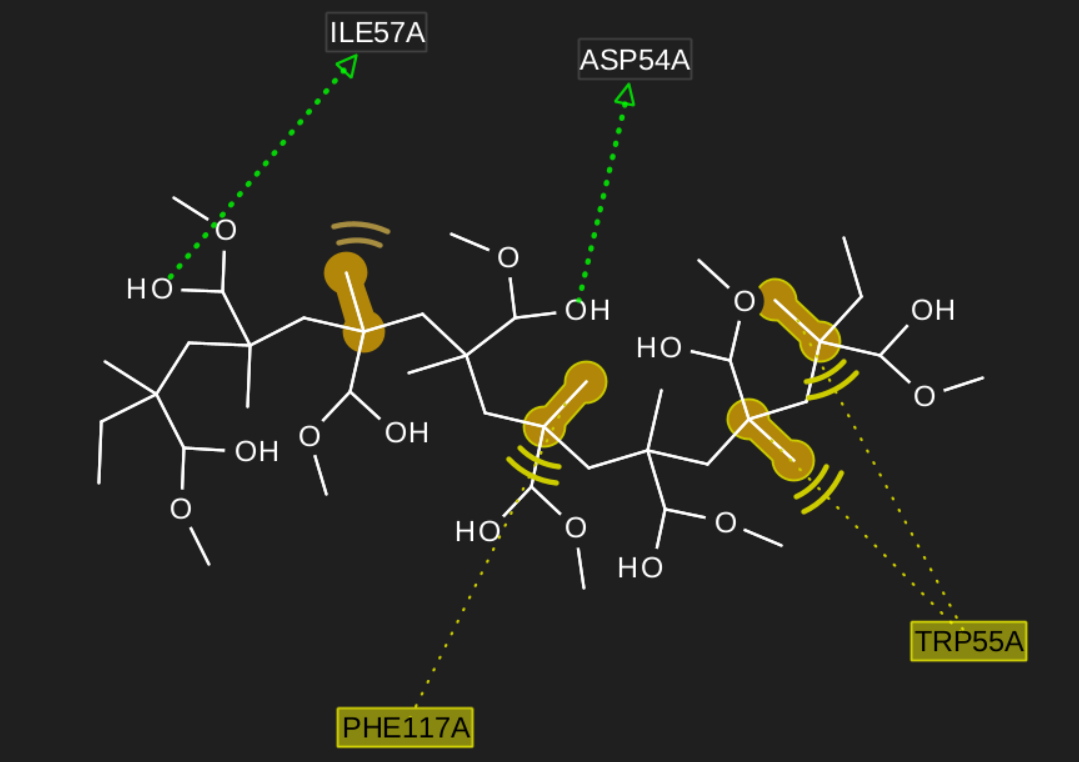 | 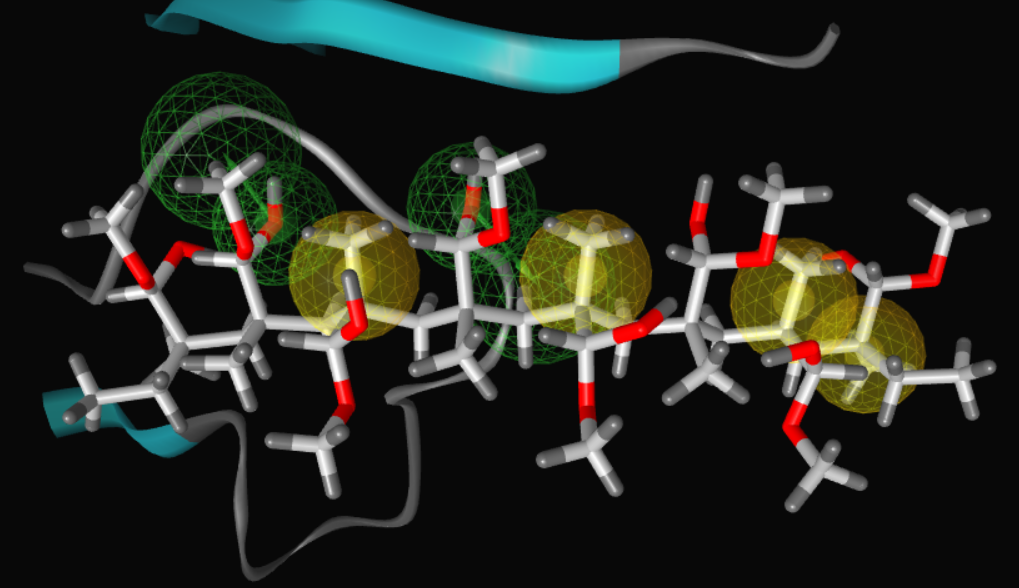 |
| BMP9:PMMA | 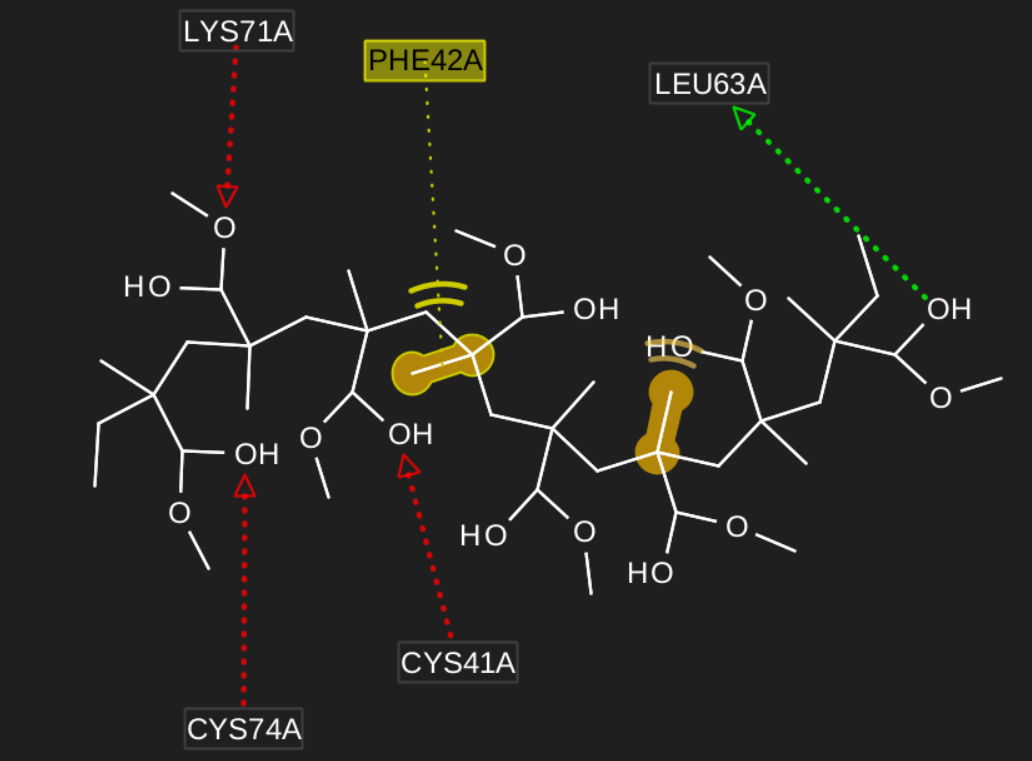 | 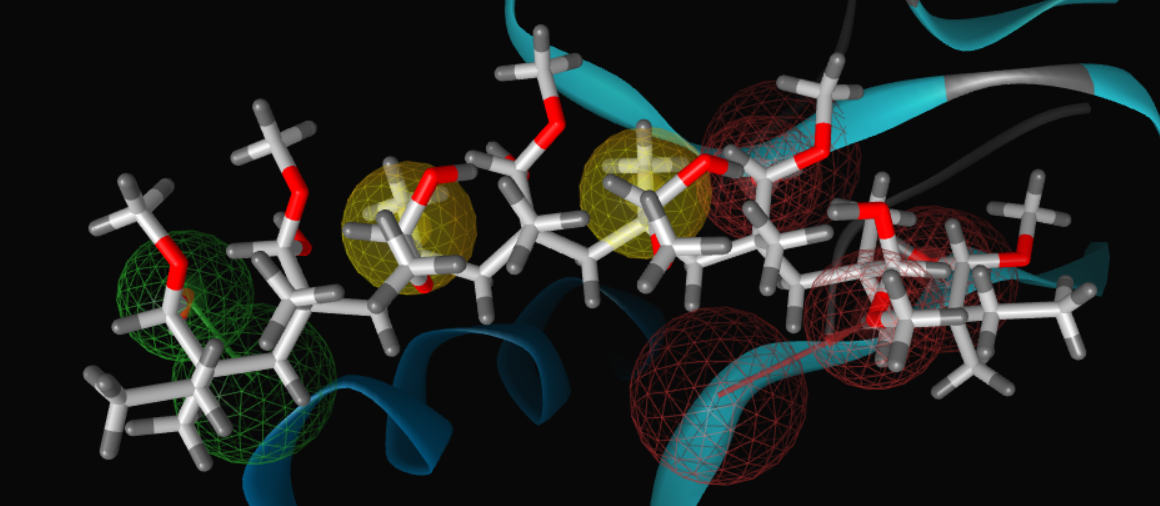 |
| COL1A1:PMMA | 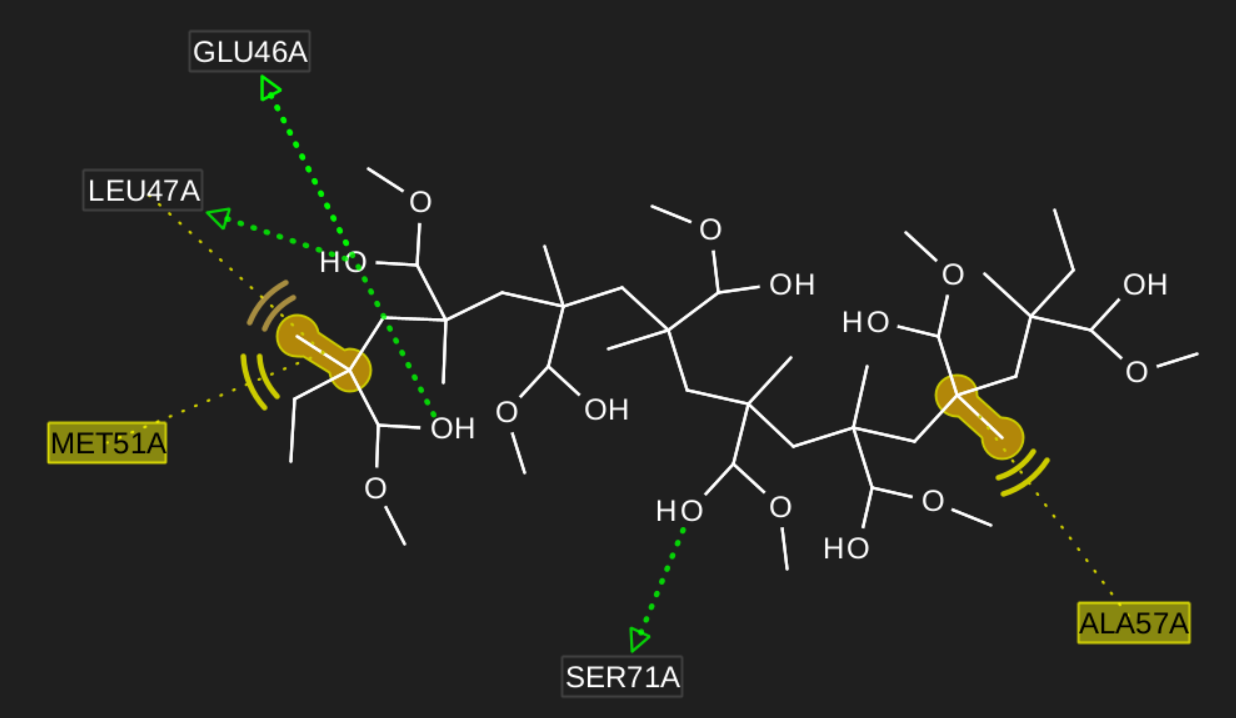 | 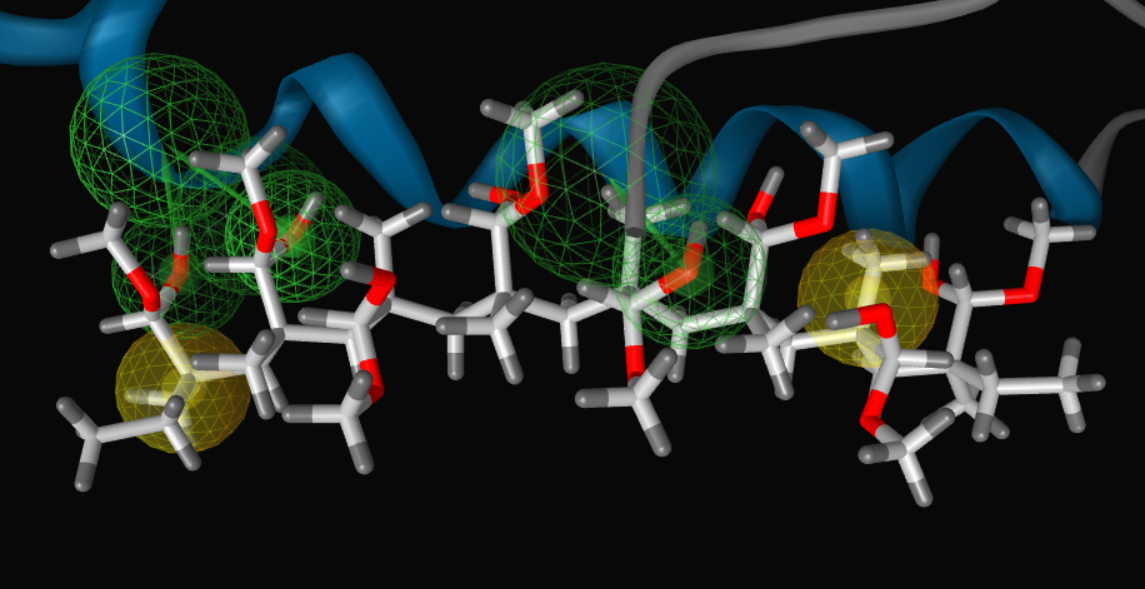 |
| DMP-1:PMMA | 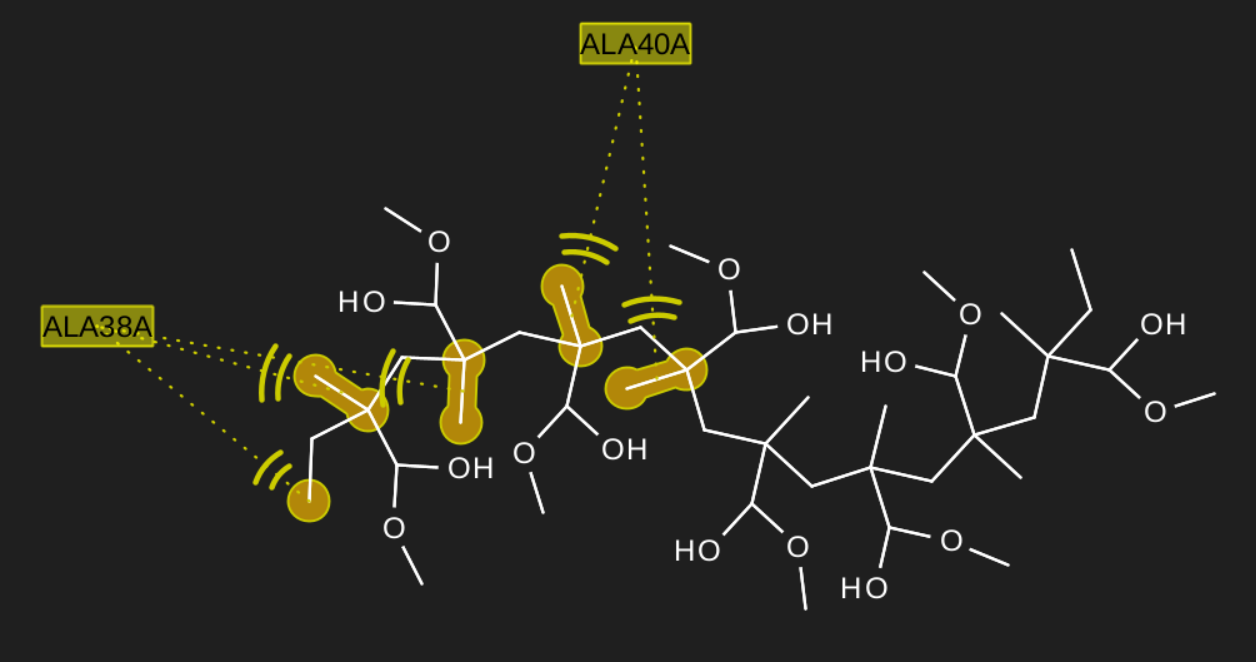 | 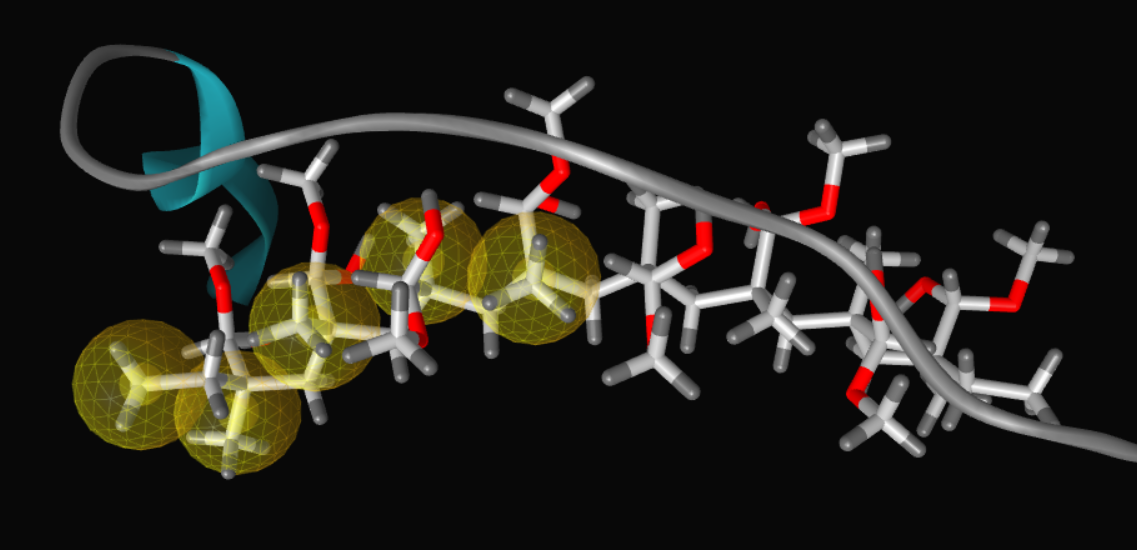 |
| Fibronectin:PMMA | 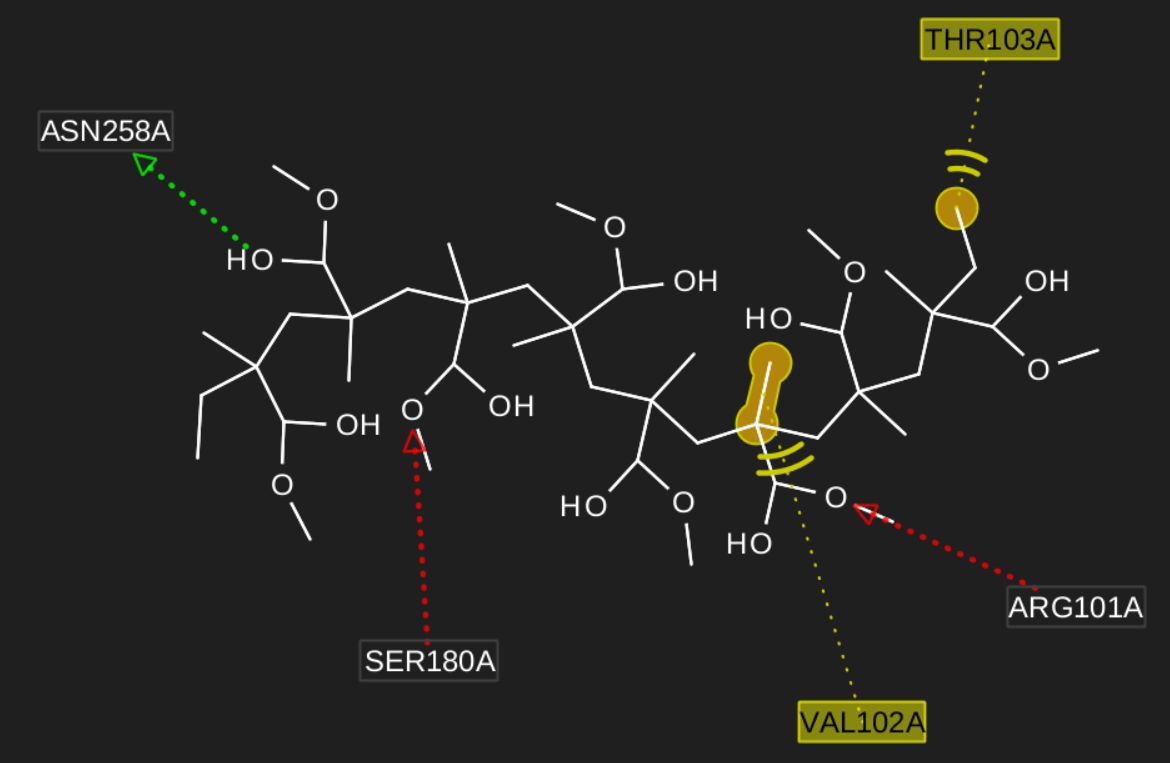 | 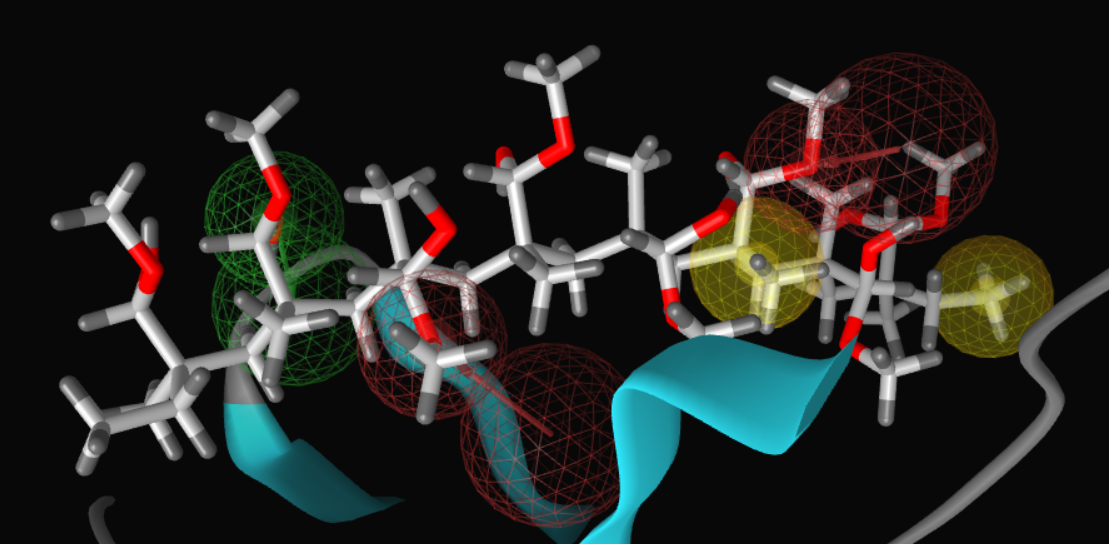 |
| IGF-1:PMMA | 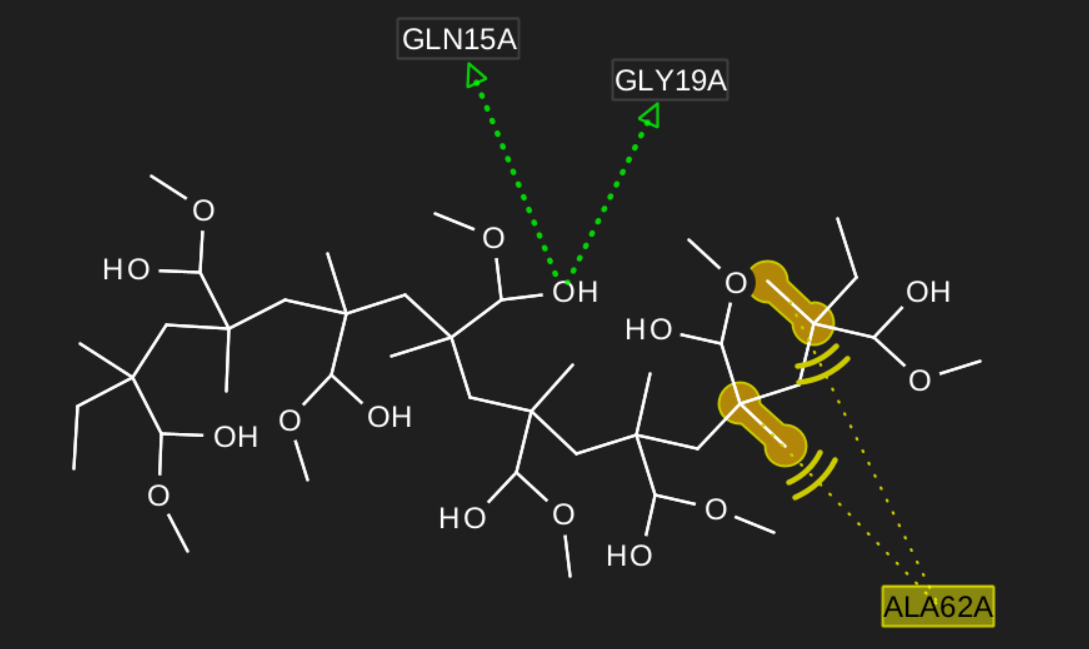 | 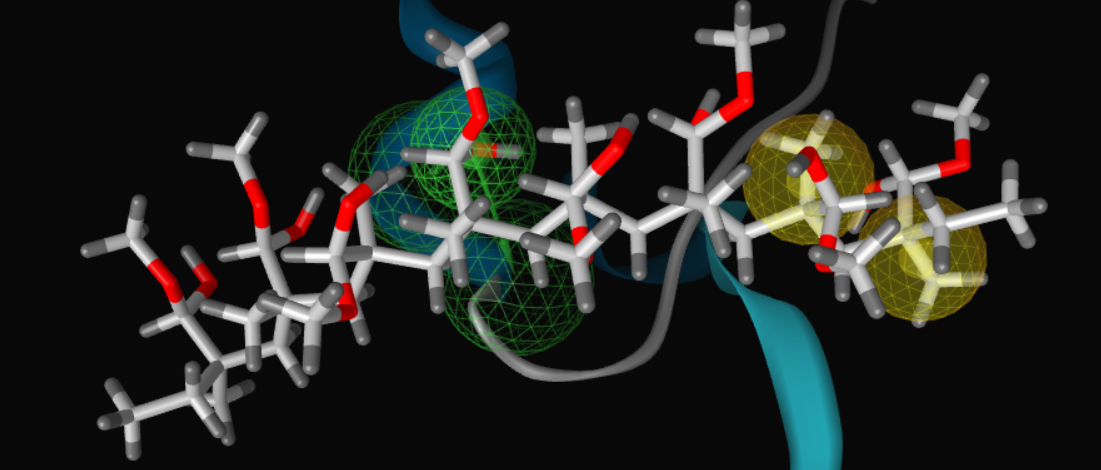 |
| NOTCH2:PMMA | 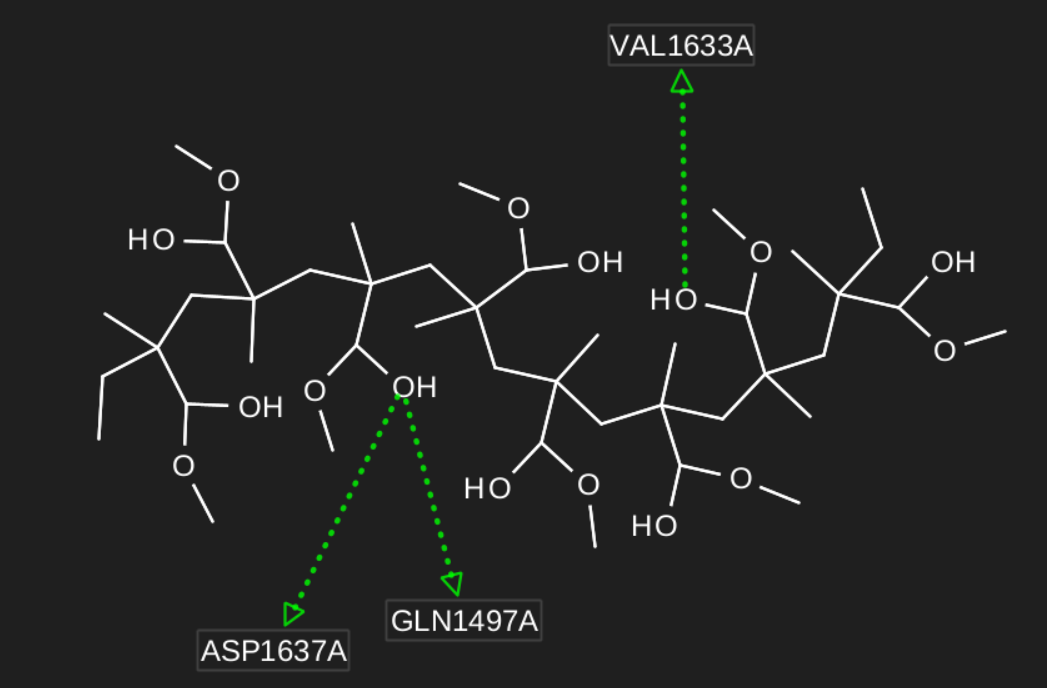 | 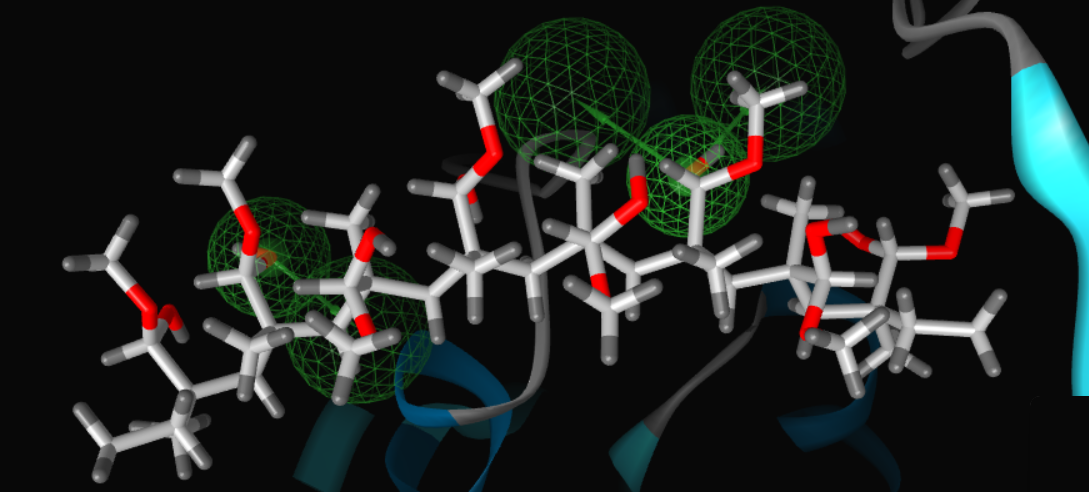 |
| Osteocalcin:PMMA | 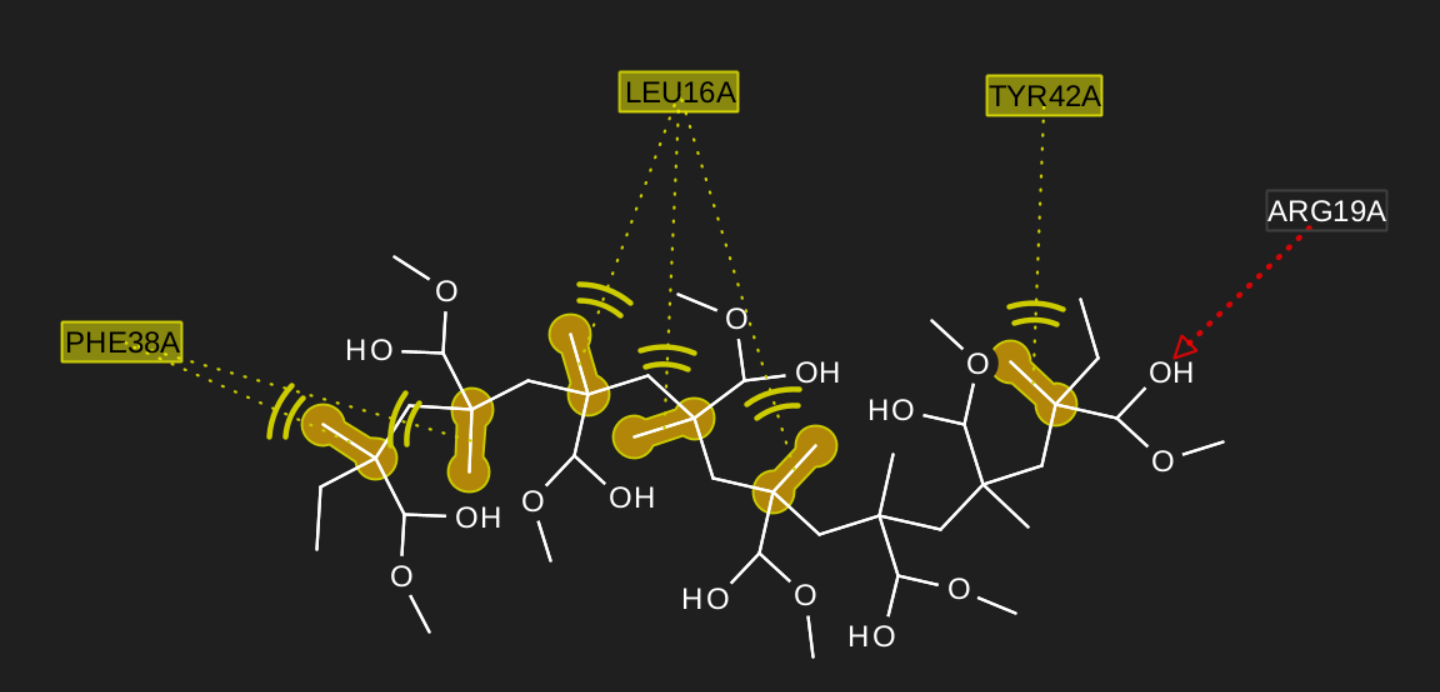 | 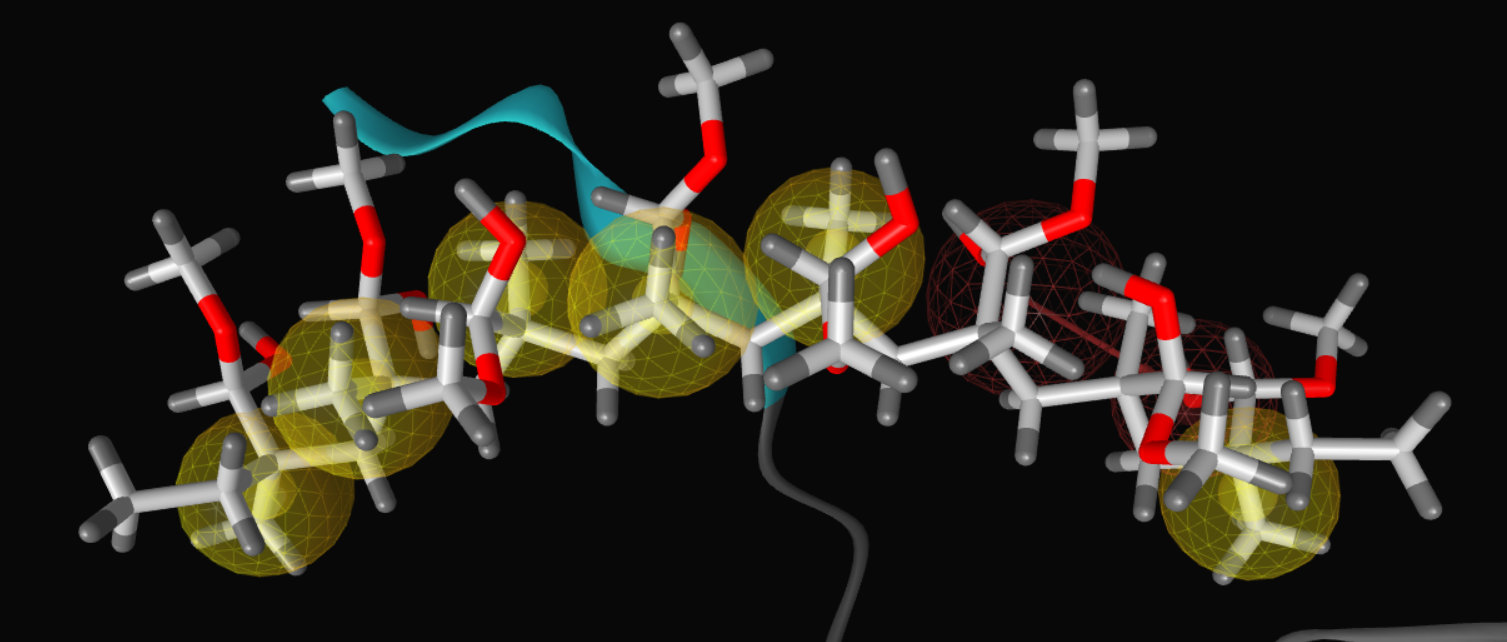 |
| Osteonectin:PMMA | 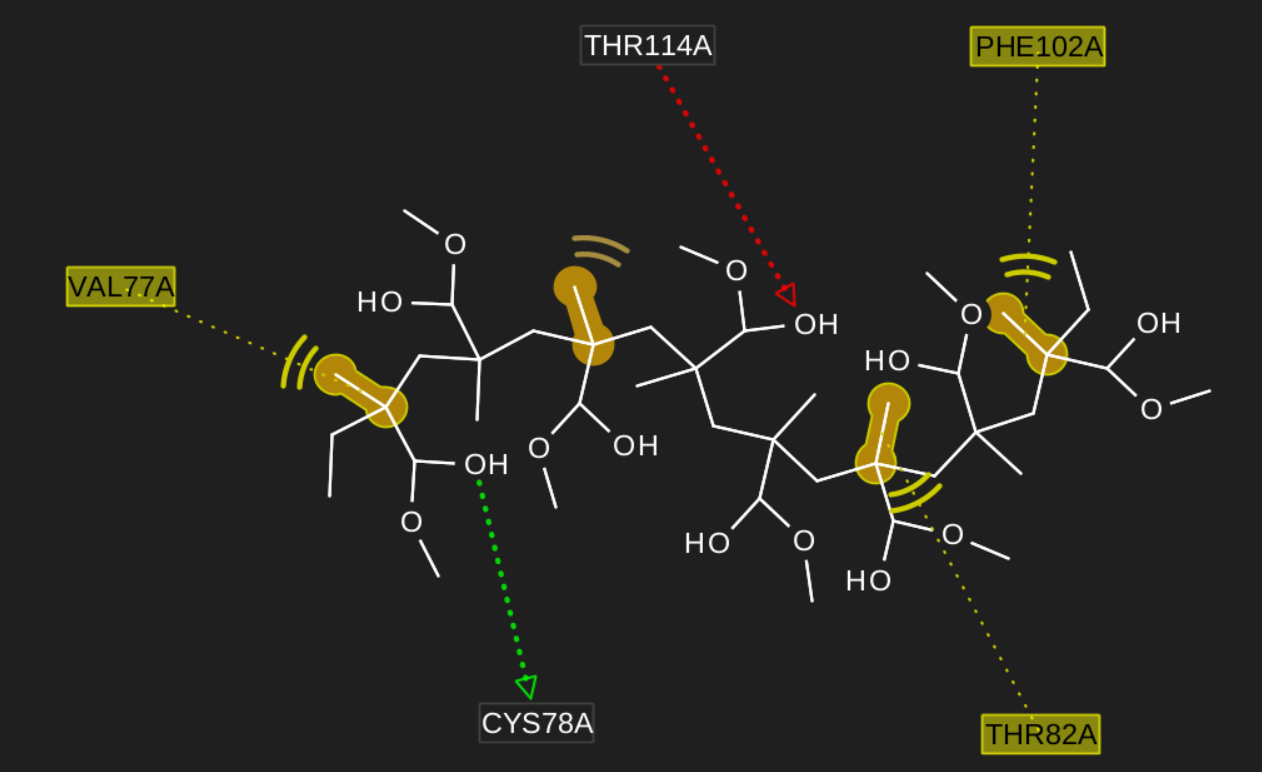 | 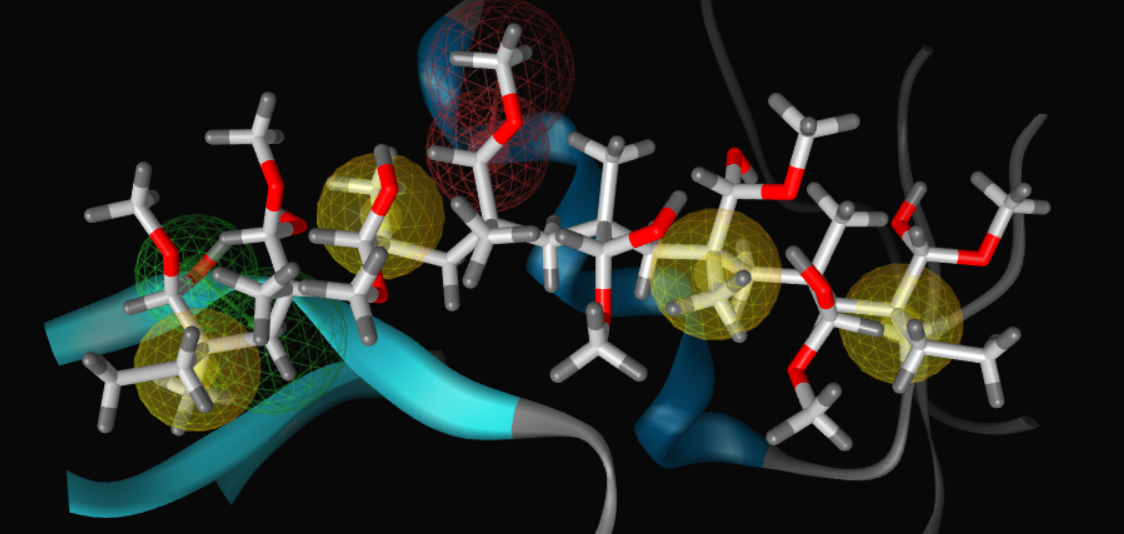 |
| Osteopontin:PMMA | 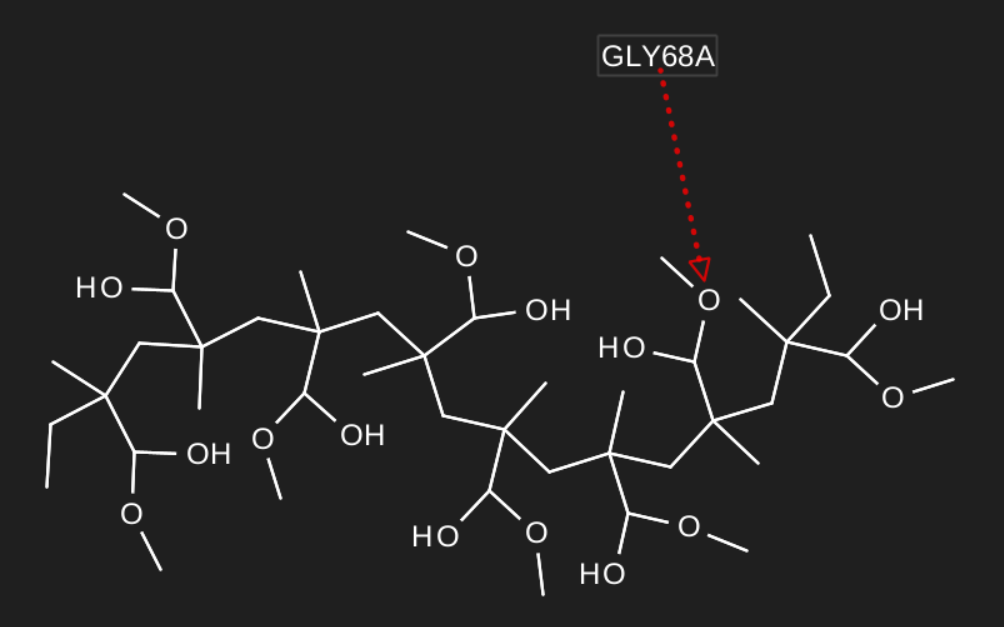 | 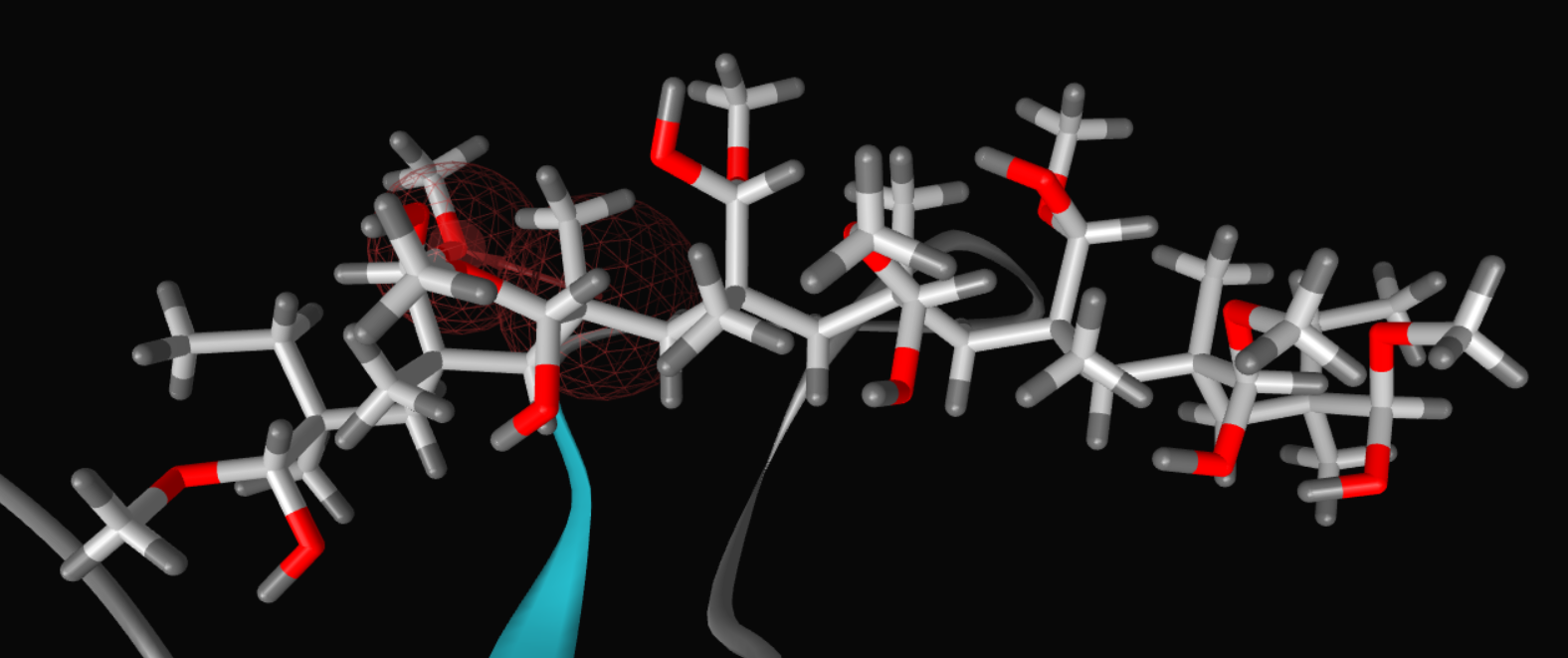 |
| Osteoprotegerin:PMMA | 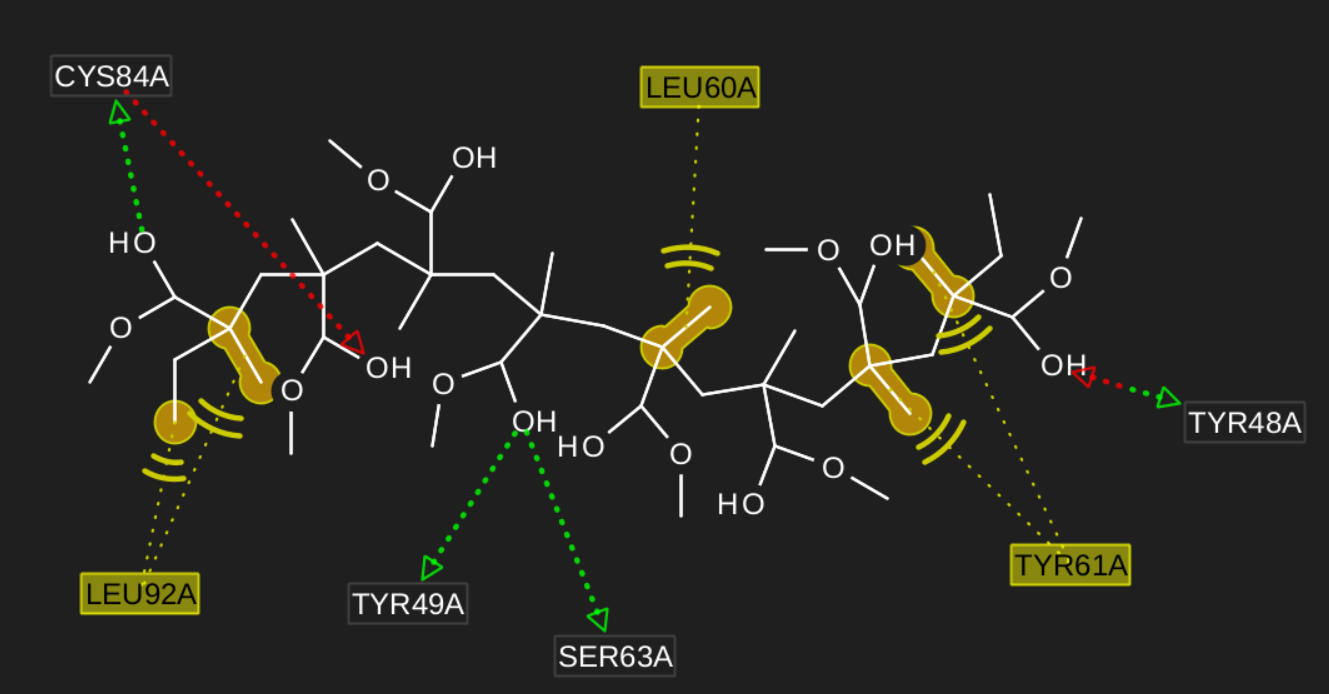 | 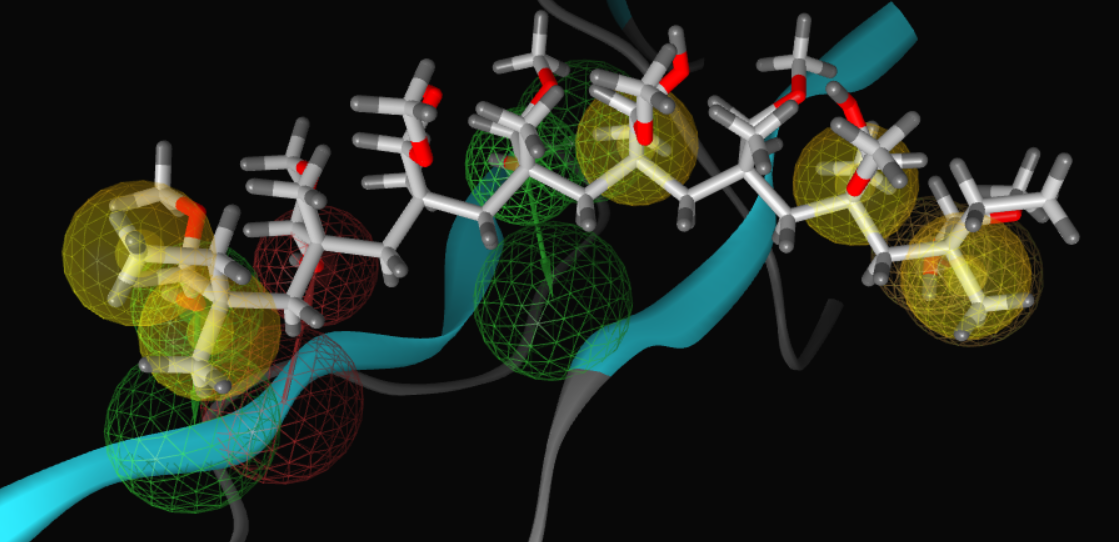 |
| Osterix:PMMA | 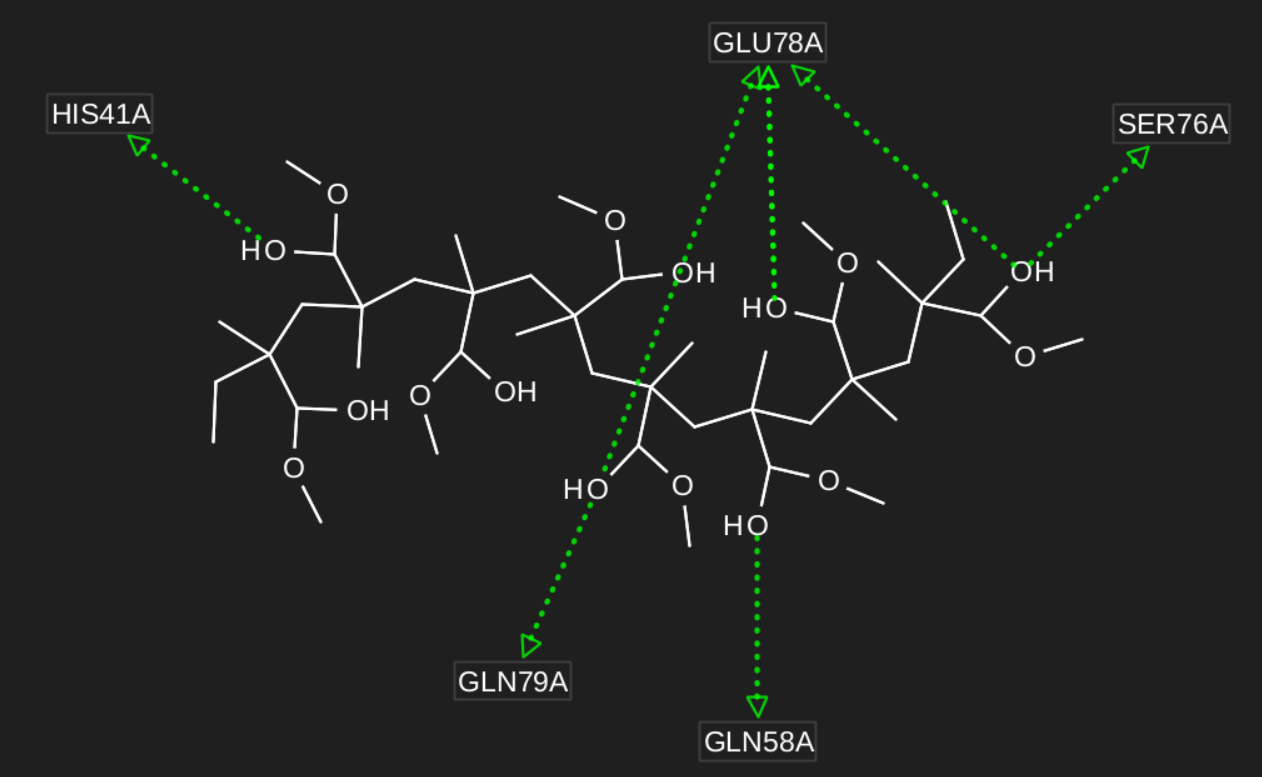 | 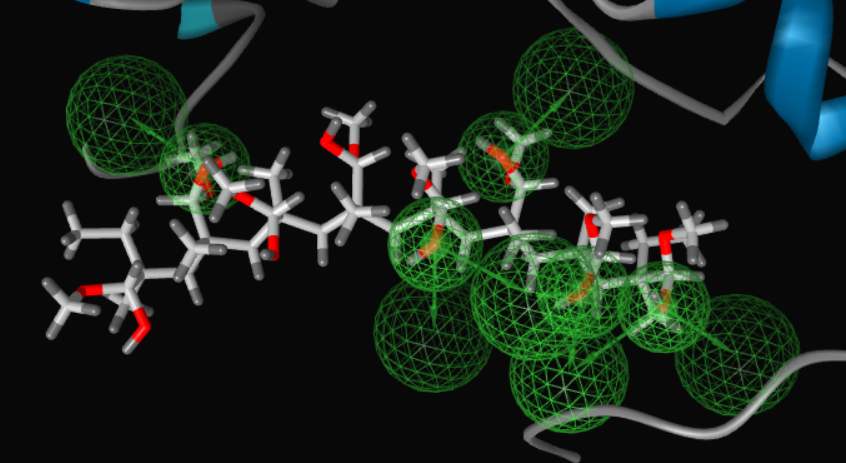 |
| RANKL:PMMA | 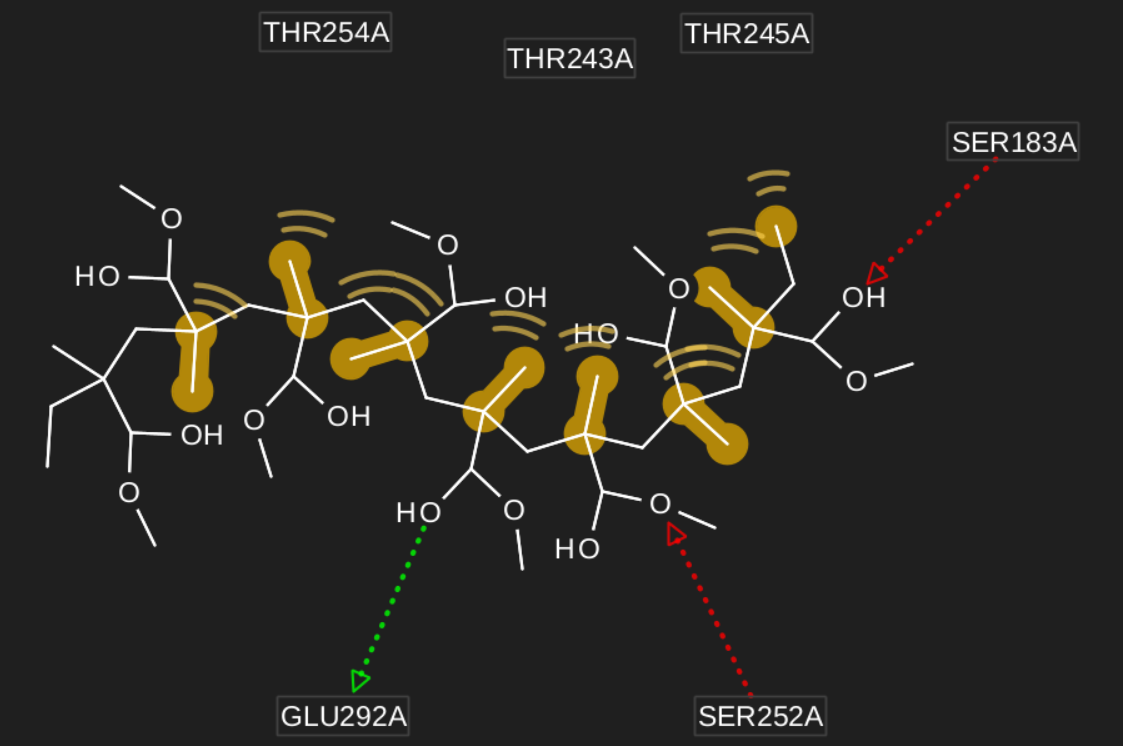 | 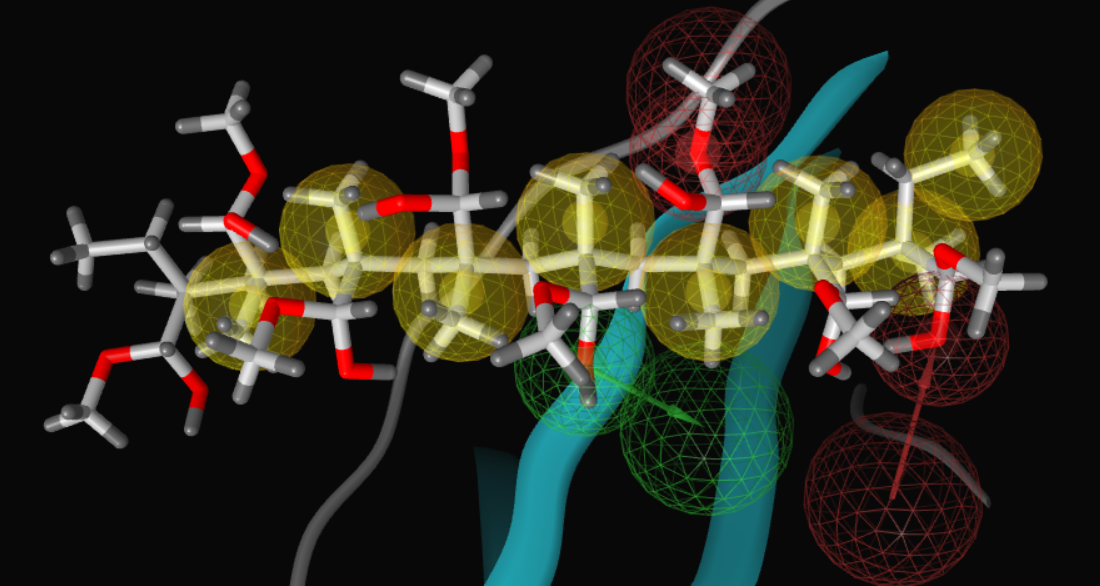 |
| RUNX2:PMMA | 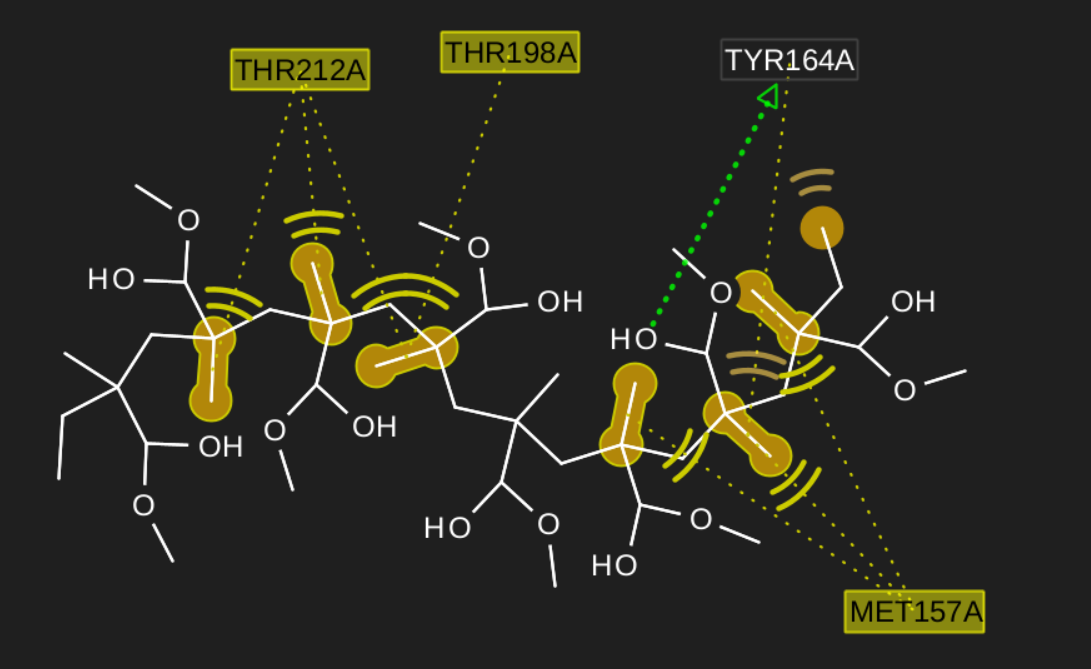 | 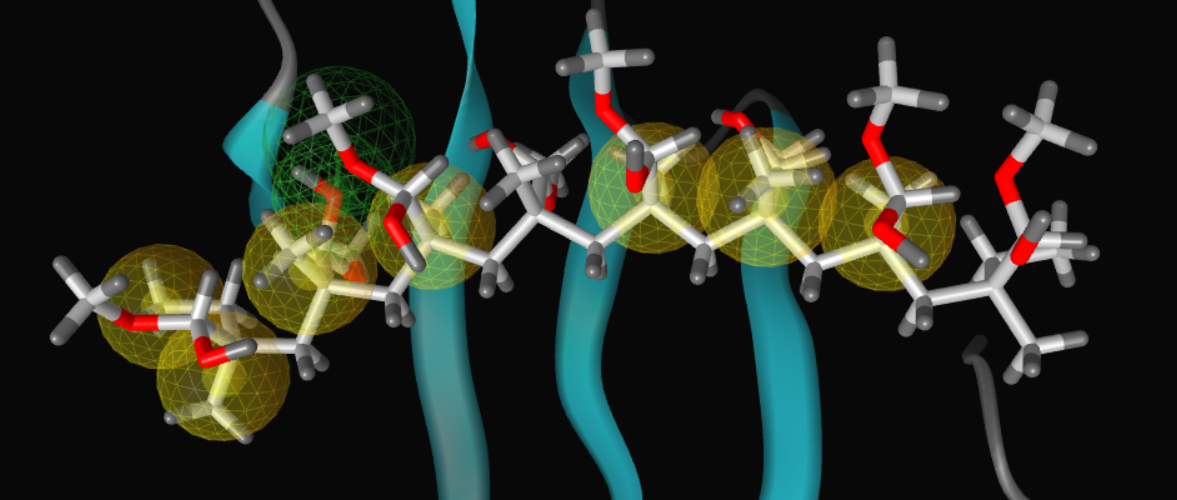 |
| TGF-B1:PMMA | 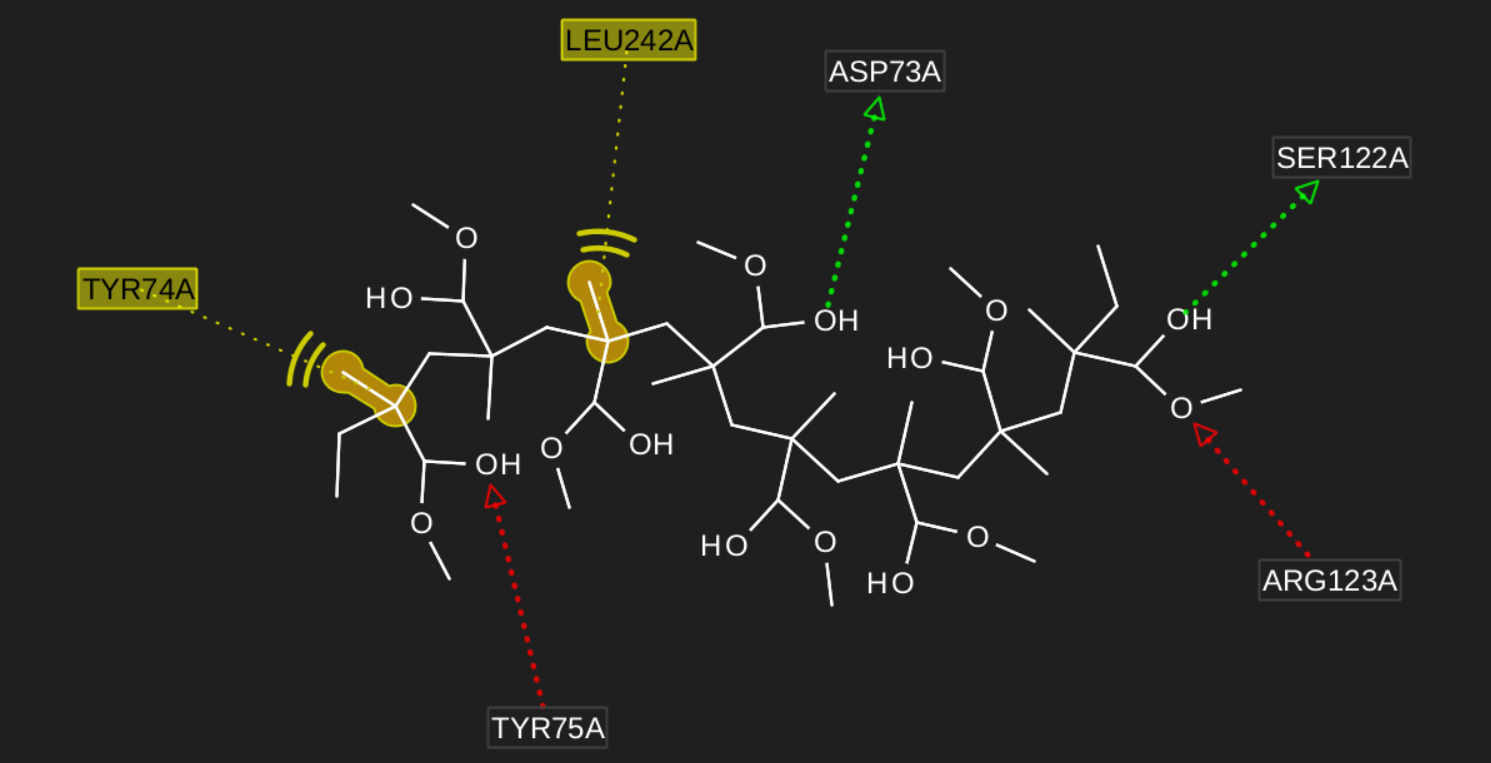 | 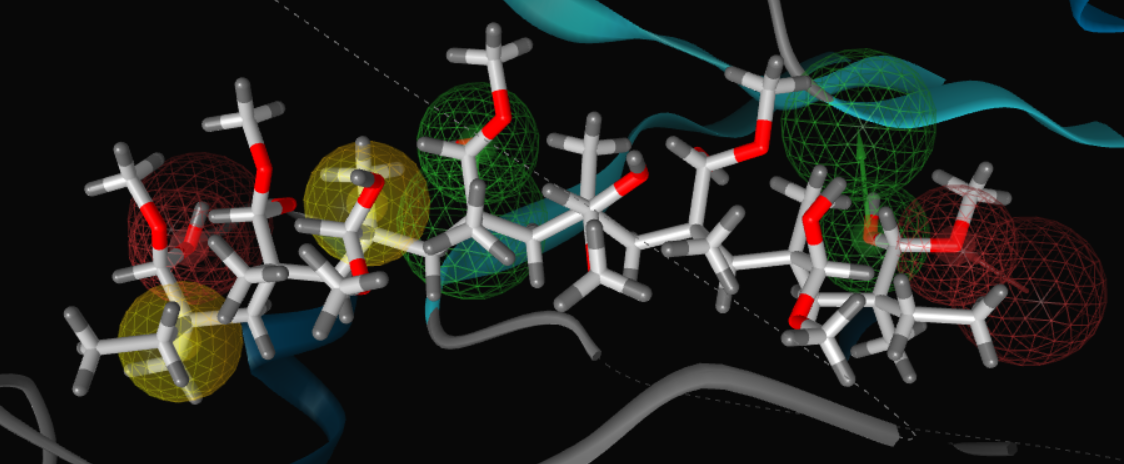 |
| TRAP:PMMA | 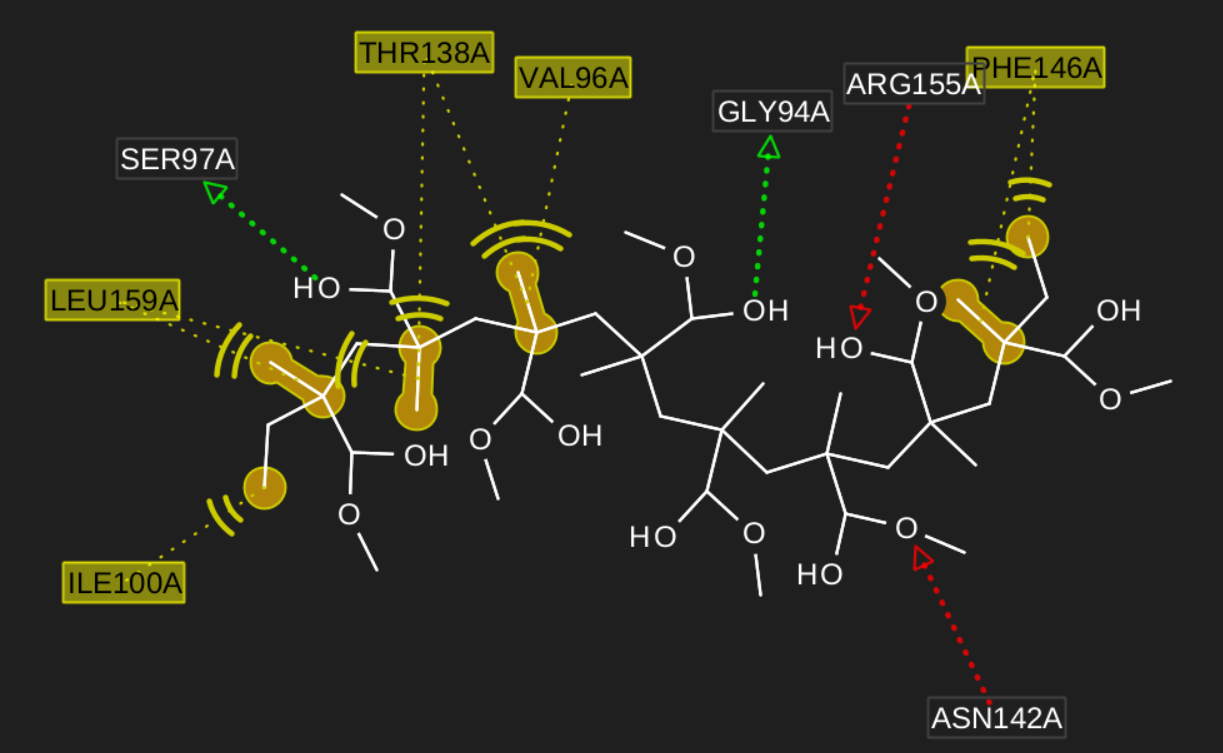 | 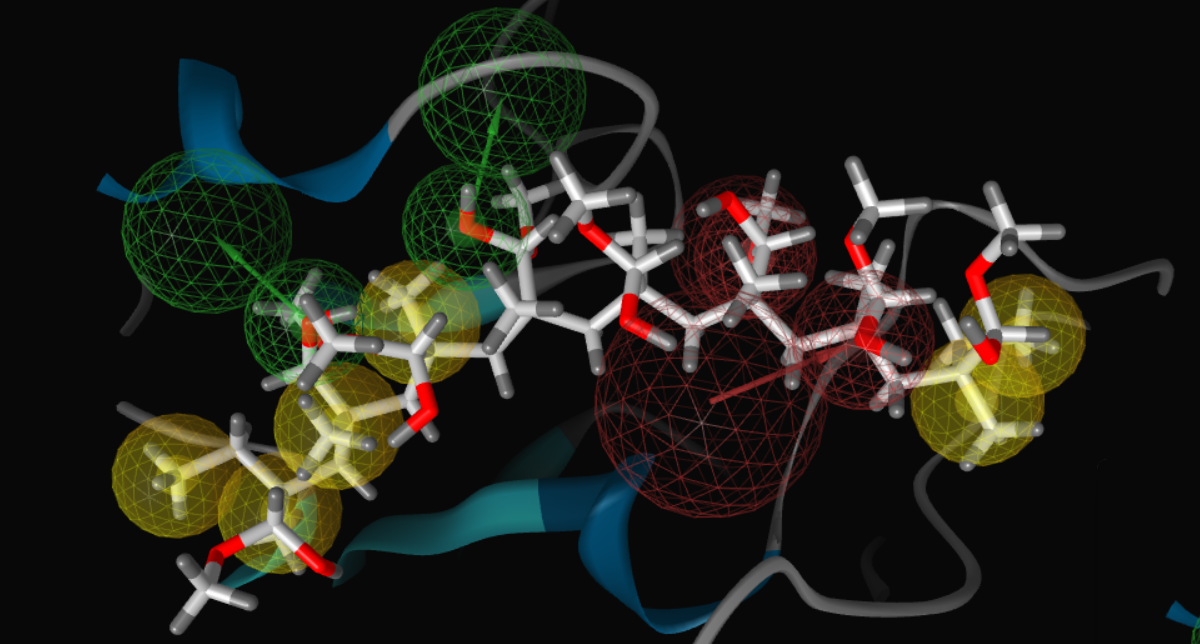 |
| Wnt3:PMMA | 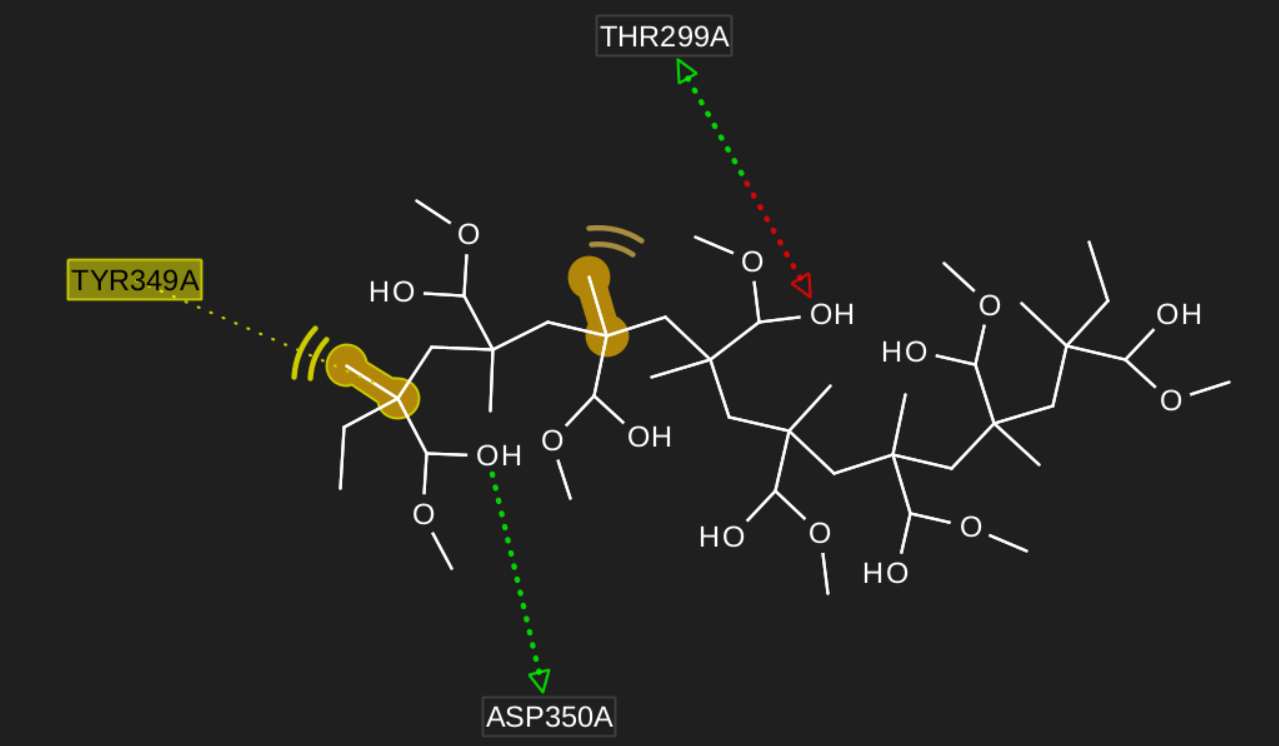 | 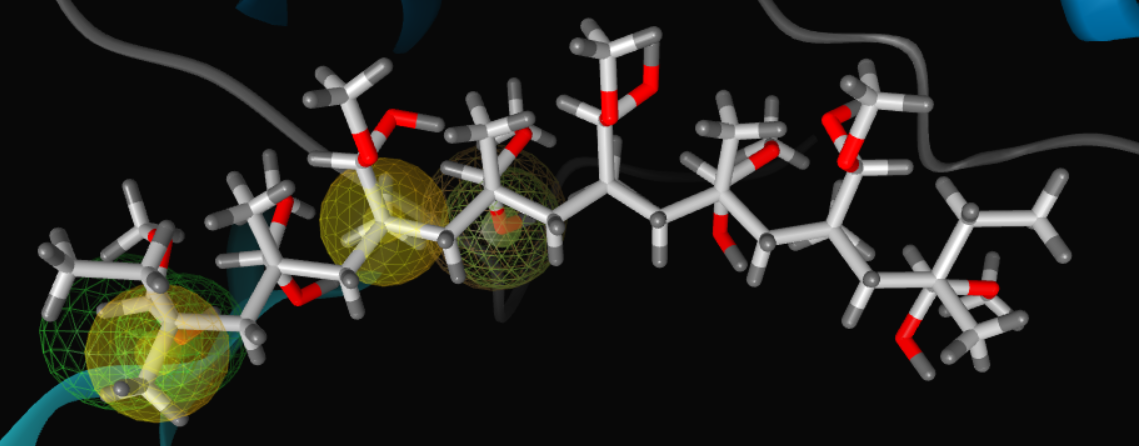 |
